# MiR-93 is related to poor prognosis in pancreatic cancer and promotes tumor progression by targeting microtubule dynamics

**DOI:** 10.1038/s41389-020-0227-y

**Published:** 2020-05-04

**Authors:** Elena Vila-Navarro, Elena Fernandez-Castañer, Maria Rovira-Rigau, Giulia Raimondi, Maria Vila-Casadesus, Juan Jose Lozano, Philippe Soubeyran, Juan Iovanna, Antoni Castells, Cristina Fillat, Meritxell Gironella

**Affiliations:** 1 Gastrointestinal & Pancreatic Oncology Group, Centro de Investigación Biomédica en Red de Enfermedades Hepáticas y Digestivas (CIBERehd)/ Hospital Clínic of Barcelona, Institut d’Investigacions Biomèdiques August Pi i Sunyer (IDIBAPS), University of Barcelona, Barcelona, Spain; 2 Gene Therapy and Cancer Group/IDIBAPS/CIBERer, University of Barcelona, Barcelona, Spain; 3 Centre de Recherche en Cancérologie de Marseille (CRCM), INSERM U1068, CNRS UMR 7258, Aix-Marseille Université et Institut Paoli-Calmettes, Parc Scientifique et Technologique de Luminy, Marseille, France

**Keywords:** Pancreatic cancer, Non-coding RNAs, Mitosis

## Abstract

Biomarkers and effective therapeutic agents to improve the dismal prognosis of pancreatic ductal adenocarcinoma (PDAC) are urgently required. We aimed to analyze the prognostic value and mechanistic action of miR-93 in PDAC. Correlation of miR-93 tumor levels from 83 PDAC patients and overall survival (OS) was analyzed by Kaplan–Meier. MiR-93 depletion in PANC-1 and MIA PaCa-2 cells was achieved by CRISPR/Cas9 and miR-93 overexpression in HPDE cells by retroviral transduction. Cell proliferation, migration and invasion, cell cycle analysis, and in vivo tumor xenografts in nude mice were assessed. Proteomic analysis by mass spectrometry and western-blot was also performed. Finally, miR-93 direct binding to candidate mRNA targets was evaluated by luciferase reporter assays. High miR-93 tumor levels are significantly correlated with a worst prognosis in PDAC patients. MiR-93 abolition altered pancreatic cancer cells phenotype inducing a significant increase in cell size and a significant decrease in cell invasion and proliferation accompanied by a G2/M arrest. In vivo, lack of miR-93 significantly impaired xenograft tumor growth. Conversely, miR-93 overexpression induced a pro-tumorigenic behavior by significantly increasing cell proliferation, migration, and invasion. Proteomic analysis unveiled a large group of deregulated proteins, mainly related to G2/M phase, microtubule dynamics, and cytoskeletal remodeling. CRMP2, MAPRE1, and YES1 were confirmed as direct targets of miR-93. MiR-93 exerts oncogenic functions by targeting multiple genes involved in microtubule dynamics at different levels, thus affecting the normal cell division rate. MiR-93 or its direct targets (CRMP2, MAPRE1, or YES1) are new potential therapeutic targets for PDAC.

## Introduction

MicroRNAs (miRNAs or miRs) are short non-coding RNAs of 18–25 bp in length that regulate gene expression at the post-transcriptional level by binding to the mRNA and causing its degradation or translational repression^1^. By exerting this function, miRs modulate a multitude of processes, such as development, cell differentiation, proliferation, apoptosis, and tumor growth^2^. Hence, miRNAs are critical tuners of transcriptional programs through the coordination of regulatory signaling networks that allow cells to adapt to microenvironmental and intracellular stress^3^. In cancer, this fine tuning is disrupted as miRNA expression is altered. Different tumors, subtypes of tumors and precursor lesions are characterized by a distinctive miRNA expression pattern^4–6^.

Pancreatic ductal adenocarcinoma (PDAC) is the fourth leading cause of cancer related deaths, as it is one of the most lethal epithelial malignancies with a very poor prognosis as over 80% of cases are diagnosed at an advanced stage of the disease and survival for these patients is less than one year^7,8^. PDAC is highly invasive, metastatic and extremely resistant to chemotherapy^9^. The standard treatment options, include surgery, chemo- and radiotherapy, and some marginally effective targeted therapies (e.g., inhibitors for VEGF, EGFR, or mTOR)^10–12^. Despite remarkable international efforts to improve the outcome of this cancer, in the majority of cases, it remains fatal^13^. Thus, a better understanding of the molecular basis of pancreatic carcinogenesis is urgently required for the identification of more effective therapeutic targets.

Currently, miRNA and anti-miR constructs are being investigated as potential therapeutic agents for cancer despite the challenges presented by the delivery of these molecules^14^. However, there is no advanced microRNA-based therapeutic approach for PDAC. To this aim, it would be important to functionally characterize particular miRNAs that seem to play relevant roles in this cancer.

This study focuses on deciphering the biological significance of miR-93 in the context of PDAC. MiR-93, a member within the miR-106b-25 cluster, paralog of the miR-17–92 cluster, has been found significantly upregulated in cancers such as gastric, lung, breast, and hepatocellular carcinoma^15,16^. Previously, we have reported that miR-93 is significantly increased in PDAC patients as well as patients with intraductal papillary mucinous neoplasm (IPMN) in relation to control individuals and presents a very high diagnostic accuracy for PDAC and IPMN cases^17,18^. In addition, miR-93 has been found differentially expressed between Low Grade Dysplasia and High Grade Dysplasia IPMNs and between IPMNs and PDACs indicating a correlation between its over-expression and progression of malignancy^17,18^.

In the present study, we identify miR-93 as an oncomiR of PDAC and shed light on the molecular mechanisms underlying the contribution of this microRNA to the pathogenesis of pancreatic cancer.

## Materials and methods

### Patients and survival analysis

For overall survival (OS) analysis, a cohort of 83 PDAC patients with long-term follow-up was obtained from Hospital Clínic of Barcelona (Supplementary Table 1). MiR-93 expression analysis by quantitative real-time polymerase chain reaction (qRT-PCR) in tumor samples was performed as previously described^17^. MiR-93 tumor levels were dichotomized into high or low using the median value of miR-93 expression as cut-off. OS was obtained for all patients from the date of diagnosis to the last contact or death and groups with high or low expression levels of miR-93 were compared by using Kaplan–Meier curves. Then, Cox regression analysis adjusted by age and gender was performed computing hazard ratio to determine the influence of high/low expression levels of miR-93 on OS. Resectable tumors refer to those considered to be entirely removed by the surgeon, mostly including stages IA, IB, and IIA. By contrast, unresectable refers to locally advanced or metastatic disease (mostly stages IIb, III, and IV). Patients who had received neoadjuvant therapy were previously excluded from the analysis. This study was approved by the Institutional Ethics Committee of Hospital Clínic of Barcelona (March 27, 2008) and written informed consent was obtained from all patients in accordance with the Declaration of Helsinki.

### Cell culture

Immortal human pancreatic duct epithelial HPDE cell line, kindly provided by Dr. F.X. Real (CNIO, Madrid, Spain), was cultured and maintained in keratinocyte serum-free (KSF) medium supplemented by epidermal growth factor and bovine pituitary extract; soybean trypsin inhibitor was used to stop trypsin effect (Gibco, Thermo Fisher Scientific Inc., Foster City, CA, USA)^19^. HEK-293T and human pancreatic tumor cell lines PANC-1 and MIA PaCa-2 were obtained from the American Type Culture Collection (ATCC, Manassas, VA, USA). HEK-293T, PANC-1, and MIA PaCa-2 were maintained in Dulbecco’s Modified Eagle’s Medium (DMEM) and supplemented with 10% fetal bovine serum, 1% penicillin–streptomycin (Gibco, Thermo Fisher Scientific Inc.). Cells were cultured in a humidified atmosphere (5% CO_2_) at 37 °C and tested for mycoplasma contamination.

### RNA extraction and quantitative RT-PCR

Total RNA was extracted from culture cells using miRNeasy Mini Kit (Qiagen, Hilden, Germany) according to the manufacturer’s protocol. For miRNA expression analysis, qRT-PCR was performed using TaqMan microRNA Assays, TaqMan miR-93-5p and RNU6B probes (Thermo Fisher Scientific Inc.) according to the manufacturer’s instructions. Briefly, 5 ng of total RNA was reverse-transcribed to cDNA under the following reaction conditions: 16 °C for 30 min, 42 °C for 30 min, and 85 °C for 5 min. RT-PCR was run on Viia7 Real-time PCR System (Thermo Fisher Scientific Inc.) at 95 °C for 10 min, followed by 50 cycles of 95 °C for 15 s and 60 °C for 1 min. All reactions were run in duplicate and miRNA data were normalized against RNU6B. Cycle threshold (Ct) was determined with fixed threshold settings using QuantStudio^TM^ Real Time PCR Software and 2^(−ΔCt)^ was calculated to obtain relative miRNA levels.

### Cell proliferation and doubling time assessment

A total of 2.5 × 10^3^ cells were seeded in quadruplicates in a 96-well plate. Cell viability was measured at 24, 48, 72, and 96 h post-seeding using the CellTilter 96^®^ Aqueous One Solution Cell Proliferation Assay, MTS (Promega Corporation, Madison, WI, USA), according to the manufacturer’s instructions. For the doubling time, cells were trypsinized and manually counted using Trypan Blue Stain (Lonza, Walkersville, MD, USA) at two different time points. Then, cell doubling time was calculated with the formula:

$$\frac{{{\mathrm{duration}}\,{\rm{x}}\,{\mathrm{log}}\left( 2 \right)}}{{\log \left( {{\mathrm{final}}\,{\mathrm{conc.}}} \right) - \log ({\mathrm{initial}}\,{\mathrm{conc}}.)}}.$$

### Cell migration and invasion assays

For the wound-healing assays, 8 × 10^5^ cells were seeded in a 6-well plate and were grown factors-starved for 24 h at full confluency. Cells were treated with mitomycin (10 μg/ml) for 1 h at 37 °C. A sterile 1.000 μl pipette tip was used to scratch the monolayer of cells to form a wound. Cells were washed with phosphate-buffered saline and cultured in complete KSF medium. Wound closure was visualized with an inverted Olympus IX51 microscope and measured using CellSens Imaging software version 1.11.

For the invasion assays, 3 × 10^4^ cells were added into the inserts of a 24-well plate BD BioCoat Matrigel Invasion Chamber (Corning Inc, Bedford, MA, USA) in triplicates. After 24 h of incubation at 37 °C, cells that had invaded were fixed, stained in a solution containing 50% methanol, 10% acetic acid, and 0.1% Coomassie brilliant blue R-250, counted and imaged using a light microscope.

### Western Blot analysis

Pierce BCA protein Assay Kit (Thermo Fisher Scientific) was used to determine protein concentration. Then, 18 μg of each protein sample was denatured and resolved by electrophoresis on 4–12% gels and transferred to polyvinylidene difluoride membranes (Novex, Thermo Fisher Scientific) by standard methods. Membranes were blocked with 5% non-fat milk in tris-buffered saline-tween buffer and incubated overnight at 4 °C with the following antibodies: anti-ITGA2 (1:5000; ab181548, Abcam, Cambridge, UK), anti-CRMP2 (1:10,000; ab129082, Abcam), anti-CDK1 (1:10,000; ab133327, Abcam), anti-CHMP4B (1:250; HPA041401, Sigma-Aldrich), anti-MAPRE1 (1:5000; ab53358, Abcam), anti-MAD2L1 (1:1000; ab97777, Abcam), anti-YES1 (1:5000; ab109265, Abcam), anti-Ki67 (1:5000; ab92742, Abcam), anti-Cyclin D1 (1:10,000; sc-6281, Santa Cruz Biotechnology, Dallas, TX, USA). Protein levels were normalized after probing the same blots with anti-GAPDH (1:10,000; ab181602, Abcam), anti-Cyclophilin (0.5 μg/ml; ab16045, Abcam) or anti-β-actin (1:1000; sc-47778, Santa Cruz Biotechnology). Proteins were detected with enhanced chemiluminescent method (Amersham Biosciences, UK) following manufacturer’s instructions.

### Supplementary materials and methods contains

Retrovirus production and HPDE infection for stable overexpression of miRNA; CRISPR/Cas9 targeting of miR-93 in PANC-1 and MIA PaCa-2 cells; Senescence assay; Binucleate assay; Transient transfection of miR-93 mimic; analysis of cell cycle by flow cytometry; time-lapse live-cell imaging; in vivo study; protein sample preparation for proteomic analysis; chromatographic and mass spectrometric analysis; protein data analysis; network analysis; luciferase reporter assays; and statistical analysis.

## Results

### High levels of miR-93 in pancreatic tumor samples are associated with poor prognosis of PDAC patients

**Fig. 1:**
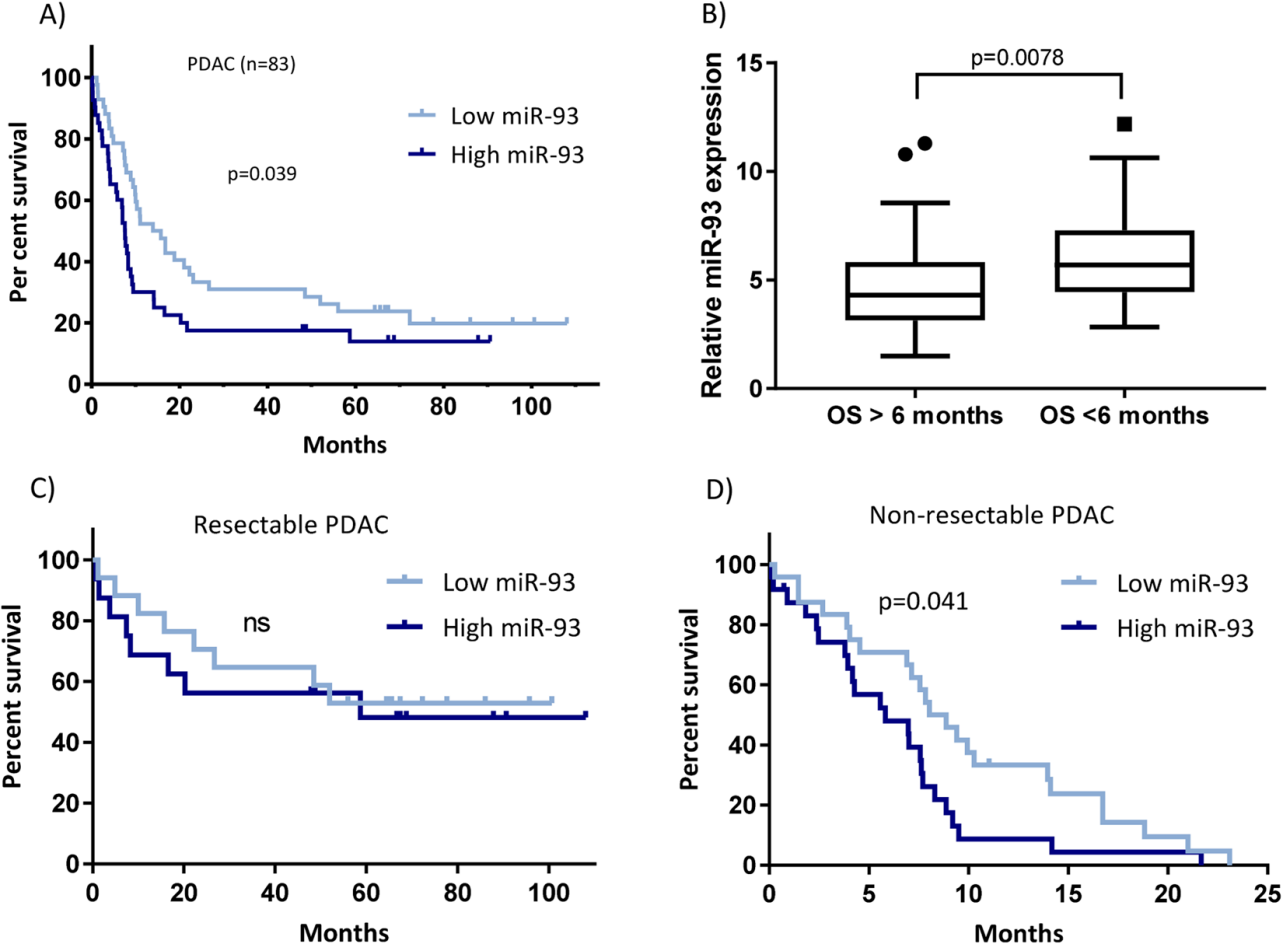
High levels of miR-93 in PDAC patients are associated with a shorter overall survival. **a** Kaplan–Meier curves for overall survival (OS) in a cohort of patients (*n* = 83) diagnosed with PDAC, according to relative expression levels of miR-93. OS showed divergent Kaplan–Meier curves according to miR-93 status. *P* values determined by log rank Mantel Cox test. **b** Relative miR-93 expression in patients with OS > 6 months compared to patients with OS < 6 months. Student’s *t* test. **c** Kaplan–Meier curves for resectable PDAC (*n* = 33) or **d** non-resectable PDAC (*n* = 48).

In our patient cohort (*n* = 83), elevated expression of miR-93 was significantly associated with a worst prognosis of PDAC (Fig. 1a, b). The median OS was 13.97 months for patients with low miR-93 expression, whereas in patients with high levels of miR-93, the median OS was 7.60 months (*p* < 0.05). Patients with an OS less than 6 months showed significantly higher tumor levels of miR-93 than patients with a higher OS (Fig. 1b). However, when we stratify these patients into resectable (*n* = 33) and unresectable (*n* = 48) tumors, only the unresectable patients maintained this significant correlation (Fig. 1c, d).

### MiR-93 depletion modifies the phenotype and behavior of pancreatic cancer cells

**Fig. 2:**
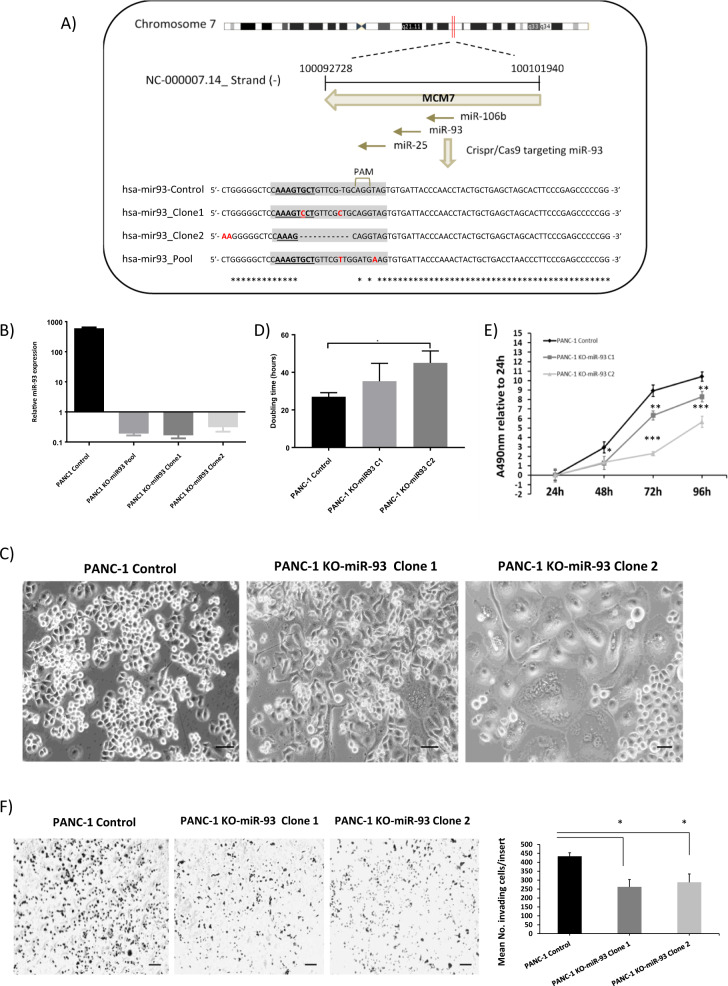
MiR-93 depletion modifies the phenotype and behavior of pancreatic cancer cells. **a** Schematic representation of the miR-106b-25 cluster and the CRISPR/Cas9 strategy for miR-93 depletion in PANC-1 cells. **b** MiR-93 expression analysis by qRT-PCR in PANC-1 control or KO-miR-93 cells, *n* = 2. **c** Optical microscope images. Scale bars 10 µm, magnitude 10×. **d** Doubling time of PANC-1 cells with or without miR-93 in hours, *n* = 3; C1: clone1 and C2: clone2. **e** MTS assay to show the effects of miR-93 depletion on PANC-1 cell proliferation and viability (*n* = 3). **f** Transwell invasion assay of PANC-1 KO-miR-93 cells compared with PANC-1 Control cells (*n* = 3). Statistics for number of invading cells per insert, representative images of the analyzed conditions magnitude 4×, scale bars 100 µm. Error bars = s.d.; *p ≤ 0.05 **p ≤ 0.01; ***p ≤ 0.001.

To decipher the biological function of miR-93 in PDAC, we performed loss-of-function experiments deleting miR-93 by CRISPR/Cas9 technology in two pancreatic cancer cell lines, PANC-1 and MIA PaCa-2. MiR-93, located in intron 13 of the host minichromosome maintenance 7 (MCM7) gene on chromosome 7q22 is co-transcribed, along with the other members of its cluster (miR-106b and miR-25) in the context of MCM7 primary transcripts. A schematic representation is detailed in Fig. 2a for PANC-1 cells. We targeted specifically the sequence of miR-93 by using the CRISPR/Cas9 system in the tumorigenic human pancreatic cell lines. From the resulting pool of cells, we generated different clones and the miR-93 gene region of all conditions was individually amplified by PCR and sequenced. As shown in Fig. 2a., small indels were introduced in the mature sequence of this miRNA, some specifically at the seed region, leading to an efficient sequence impairment. We confirmed by qRT-PCR that these indels and single nucleotide mutations resulted in depletion of miR-93, totally abolishing the production of mature miR-93 as its expression was almost undetectable as shown in Fig. 2b. We were able to knockout miR-93 without affecting the expression of miR-106b, miR-25, or MCM7 (Supplementary Fig. 1). The abolition of miR-93 drastically altered the phenotype of pancreatic cancer cells. PANC-1 KO-miR-93 cells were much bigger in size and acquired a different cellular morphology in comparison with PANC-1 control cells (Fig. 2c). Doubling time and proliferation curves reported a significantly slower proliferation rate of genetically modified PANC-1 cells, as shown in Fig. 2d, e. Indeed, PANC-1 KO-miR-93 clone 2 cells took almost twice the time required for PANC-1 control cells to double. Furthermore, PANC-1 KO-miR-93 clones showed a significant reduction of invasion with respect to control cells Fig. 2f.

Similar alterations were also observed in the other miR-93 KO pancreatic cancer cell line model generated in MIA PaCa-2 cells, in which a change in cellular morphology and size, and a significant reduction in cell proliferation and invasion were observed in both MIA PaCA-2 KO-miR-93 studied clones (Supplementary Fig. 2).

In order to explain this phenotype, we first tested if cells without miR-93 entered in a senescence state that could explain this phenotype, but they did not show any differences in β-galactosidase activity when compared with control cells, discarding that they could enter a state of permanent growth arrest (data not shown).

### MiR-93 depletion alters the cell cycle of pancreatic cancer cells

**Fig. 3:**
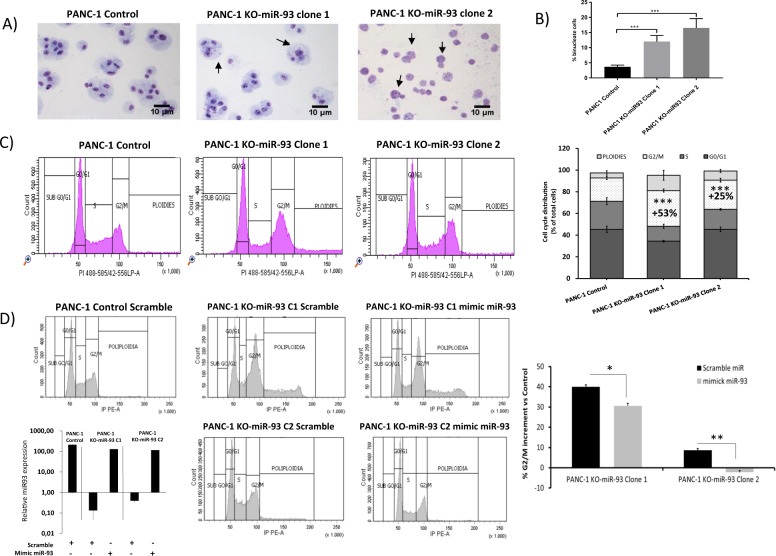
PANC-1 cells lacking miR-93 expression present an arrest at G2/M. **a**, **b** Binucleate assay: **a** Giemsa staining; representative optical microscope images, magnitude 20×. **b** % number of binucleate cells for 200 nuclei counted; *n* = 3; **c** Cell cycle analysis by flow cytometry, right: cell cycle distribution of analyzed cells, percentage. 10,000 singlets (*n* = 3). Left: a representative image of each condition is shown. **d** Cell cycle analysis for phenotype rescue experiments. Left: representative images of each condition are shown. C1: clone 1; C2: clone 2. Totally, 10,000 singlets, (*n* = 3). Down: qRT-PCR expression of miR-93 after miR-93 mimic transfection vs. scramble. Right: effects of miR-93 overexpression in PANC-1 KO-miR-93 cells represented as the percentage of increase in G2/M cells with respect to PANC-1 control cells transfected with scramble miR in both KO-miR-93 clones transfected with scramble or miR-93 mimick. Error bars: s.d. *p < 0.05 **p < 0.01 ***p < 0.001.

We observed by light microscopy that some PANC-1 KO-miR-93 cells had multiple nuclei. To confirm this observation, we performed a binucleate assay where cells were fixed and stained. Indeed, a higher occurrence of multinucleated cells, with two or more nuclei per cell was significantly found in PANC-1 KO-miR-93 cells (Fig. 3a, b). Consistently, time-lapse recordings show an alteration of the cell cycle rhythm as significantly less cell divisions were observed in PANC-1 KO-miR-93 cells during 15 h of recording in comparison to PANC-1 control cells. Representative recordings where 8 cell divisions during a 15-h period can be observed in PANC-1 control cells whereas no cell division is appreciated in PANC-1 KO-miR-93 cells during the same period are shown in Supplementary Figs. 3 and 4, respectively.

Concordantly, we analyzed the effects of miR-93 deficiency on the cell cycle of PANC-1 cells by flow cytometry. We observed that lack of miR-93 induced a significant increase of cells arrested at G2/M phase with respect to controls, a 53% and a 25% increase in clone 1 and clone 2, respectively (Fig. 3c). Moreover, an increase in the percentage of polyploid cells in both KO-miR-93 clones is also shown in Fig. 3c, inferring a possible implication of miR-93 in this phase of the cell cycle. To further corroborate this hypothesis, we tried to recover the G2/M arrested cells in PANC-1 KO-miR-93 clones by overexpressing miR-93 through transient miR-93 transfection. In this experiment, under transfection conditions, miR-93-deleted PANC-1 cells showed a 40% and 8.8% increase in the percentage of G2/M cells for clone 1 and 2, respectively, vs. PANC-1 control cells transfected by a scramble miR. Overexpression of miR-93 in PANC-1 KO-miR-93 cells induced a significant reduction of this G2/M arrest, as shown in Fig. 3d. In clone 1, a 24% recover was observed whereas miR-93 transfection in clone 2 totally recovered the G2/M arrest induced by miR-93 depletion.

### MiR-93 deficiency impairs xenograft tumor growth in mice

**Fig. 4:**
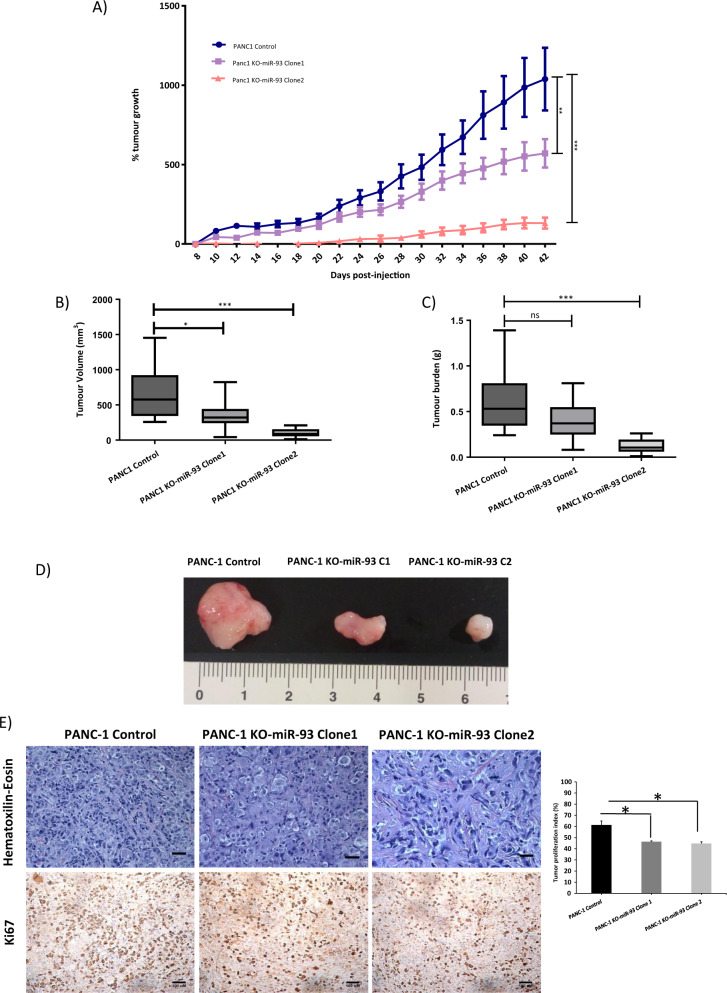
In vivo experiments demonstrate that deficiency of miR-93 generates smaller heterotopic human pancreatic xenografts with a significant slower progression. **a** Tumor growth (%) measurement every two days, from day 8 after subcutaneous transplantation of PANC-1 control or KO-miR-93 cells into each flank of 6-weeks-old nude mice to day 42 (*n* = 14 tumors per group). **b** Tumor volume (mm^3^) 7 weeks postinjection and **c** tumor burden (g) 7 weeks postinjection. **d** Representative images of tumors at 7 weeks postinjection (*n* = 14 tumors per group). **e** Hematoxilin-eosin and Ki67 staining of fixed tumors; left: representative optical microscope images (*n* = 14), hematoxilin-eosin: magnitude 20×, scale bars: 50 µm, Ki67: magnitude 10×, scale bars:100 µm; right: tumor proliferation index, Ki67 positive cells with respect to total cells (*n* = 3) C1: clone1 and C2: clone 2. Error bars = s.d. *p ≤ 0.05; **p ≤ 0.01; ***p ≤ 0.001.

We further examined the effects of miR-93 deficiency in vivo by inoculation of human pancreatic cancer cells (control PANC-1, PANC-1 KO-miR-93 clone1, C1, or clone2, C2) subcutaneously into each flank of 21 immunodeficient mice, a total of 42 tumors (*n* = 14 per group). We assessed the capability of these cells to generate heterotopic pancreatic tumors. PANC-1 KO-miR-93 (C1 and C2) cells developed significantly smaller tumors that had a slower growth rate than control cells (Fig. 4a). Specifically, tumors from C2 were either very small with a slow progression or totally undetectable, even after 42 days post-cell injection. These results suggest that miR-93 promotes tumor growth. In addition, after tumor growth follow-up, we measured and weighed the tumors on necropsy. Tumors from control PANC-1 cells were significantly bigger in size and more vascularized than tumors from PANC-1 KO-miR-93 (C1 and C2) (Fig. 4b, d) and had a significantly higher tumor burden than tumors from C2 (Fig. 4c). A representative image of tumors from each group (*n* = 14) at 7 weeks postimplantation is depicted in Fig. 4d. Hematoxylin–eosin staining of the tumors showed that PANC-1 KO-miR-93 tumors had enlarged cells in comparison to those from control PANC-1 tumors and appeared more fibrotic and less vascularized than control tumors. Concordantly, Ki67 staining showed a decrease on the number of proliferating cells in KO-miR-93 tumors accompanied by a statistically significant reduction of the tumor proliferation index (Fig. 4e).

### Overexpression of miR-93 confers pro-tumorigenic characteristics to normal pancreatic ductal cells

**Fig. 5:**
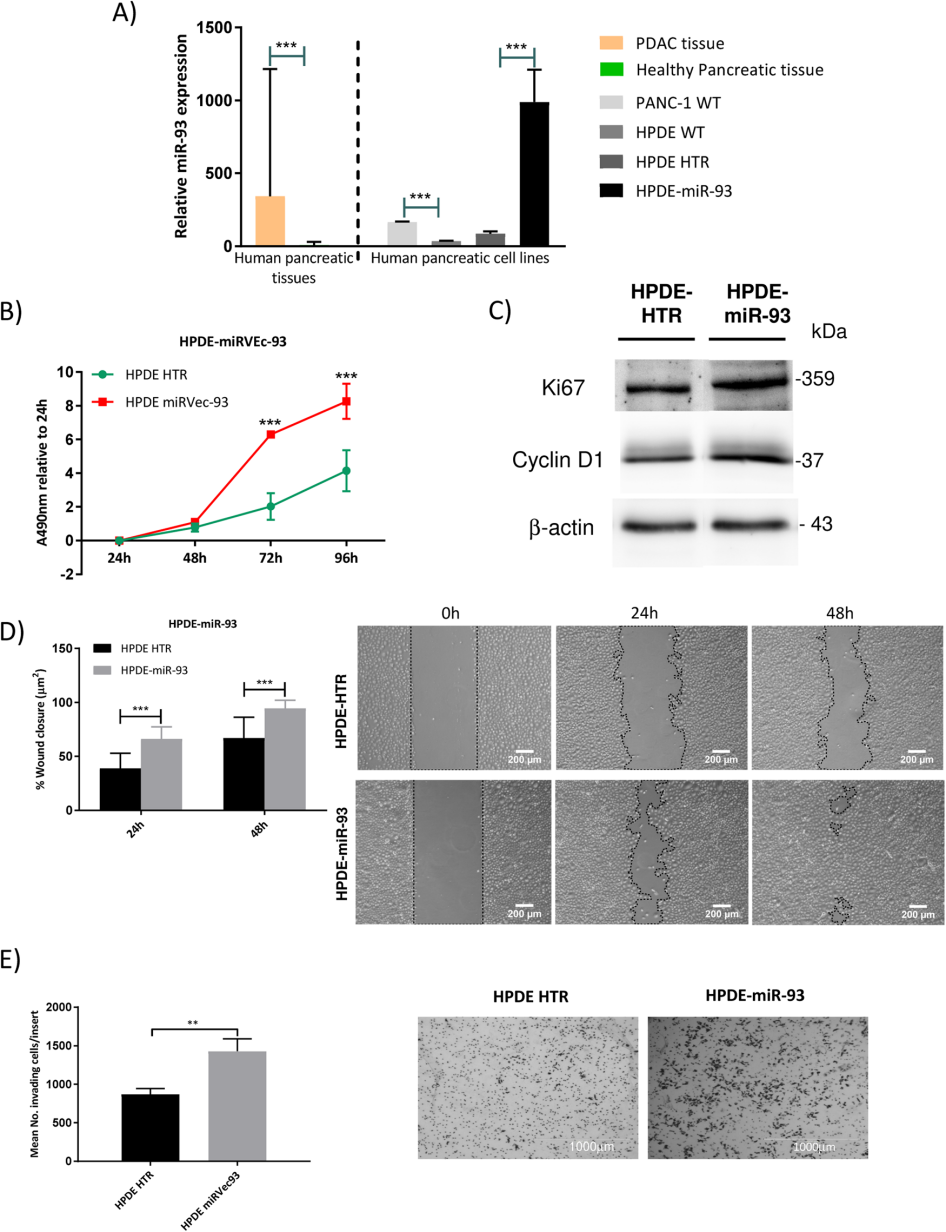
HPDE overexpressing miR-93 proliferate, migrate, and invade more than control cells. **a** MiR-93 expression in pancreatic tissue samples and pancreatic cell lines. PDAC tissues (*n* = 50), healthy pancreatic tissues (*n* = 25) and PANC-1 (*n* = 3) and HPDE (*n* = 3) pancreatic cell lines. MiR-93 expression normalized against RNU6B. **b** MTS assay to assess the effects of miR-93 overexpression on cell viability (*n* = 4). **c** Western Blot of Ki-67 and Cyclin D1 normalized against β-actin on HPDE-hTR and HPDE-miR-93 protein extracts (representative image of *n* = 3). **d** Wound healing assay to assess the effects of miR-93 on cellular motility by calculating per cent wound closure over time points (*n* = 11). Representative images are shown. **e** Transwell invasion assay of HPDE-miR-93 expressing cells compared with HPDE-hTR control cells (*n* = 3). Representative images are shown. Error bars = s.d.; **p ≤ 0.01; ***p ≤ 0.001.

Expression levels of miR-93 are significantly higher in PDAC tissues than in healthy pancreatic tissues, although heterogenous. MiR-93 expression in human pancreatic ductal epithelial (HPDE) cells are similar to healthy human pancreatic tissues and significantly lower than in the tumorigenic pancreatic cancer cell line PANC-1, thus HPDE cells were used to stably overexpress miR-93 in order to carry out gain of function analysis (Fig. 5a).

When cell viability was assessed at 72- and 96-h post-seeding, HPDE-miR-93 overexpressing cells proliferated significantly faster than control HPDE-hTR cells (Fig. 5b). Concordantly, HPDE-miR-93 cells showed higher protein levels of proliferation markers Ki-67 and Cyclin D1 (Fig. 5c). Moreover, miR-93 overexpression significantly increased cellular motility and invasion as these cells were faster in closing an artificial gap and invaded significantly more than control HPDE-hTR cells (Fig. 5d, e).

### Proteomic analysis unveils a large group of significantly dysregulated proteins resulting from modulating the expression of miR-93

MiRNAs exert their function by modulating protein translation of their targets. A full understanding of miRNA function requires knowledge of their targets. To identify potential miR-93 targets potentially involved in pancreatic carcinogenesis causing some of the above mentioned observed effects, we performed two different proteomic assays. One with PANC-1 cells (*n* = 3 control; *n* = 3 KO-miR-93) and another with HPDE cells (*n* = 3 control hTR; *n* = 3 miR-93). From the PANC-1 proteomic analysis, a total of 385 proteins were altered: 131 proteins were differentially expressed between control and KO-miR-93 cells (fold change (control vs. KO) ≤ −0.5; *p* value ≤ 0.05 and protein detection in 2 or 3 sample for each condition) and 254 proteins were undetectable in control cells and only present in KO-miR-93 cells (Supplementary Tables 2 and 3). These data were further explored by the Ingenuity Pathway Analysis (IPA). IPA revealed that the most highly represented diseases/disorders with a higher number of dysregulated proteins by miR-93 depletion were cancer and organismal injury and abnormalities (Supplementary Table 4). Top networks included: (1) cellular compromise, cell cycle, cellular assembly, and organization (score = 43) and (2) cellular development, cellular growth and proliferation, cell morphology (score = 39) (Supplementary Table 4), consistent with the aforementioned observations. IPA revealed a list of significant canonical pathways for this data set, many of which are involved in the cell cycle regulation (Supplementary Fig. 5).

Regarding the proteomic analysis performed in HPDE cells, a total of 226 proteins were altered: 75 proteins were found to be significantly differentially expressed between control and miR-93 overexpression (fold change (control hTR vs. miR-93) ≥ 0.5, *p* value ≤ 0.05 and protein detection in 2 or 3 sample for each condition) and 151 proteins were only detected in control HPDE-hTR and not detected in HPDE-miR-93 cells (Supplementary Tables 5 and 6). After IPA pathway and global functional analysis, we identified that the top and second diseases/disorders related to the 226 dysregulated proteins were involved in cancer and gastrointestinal disease (Supplementary Table 7) and the top networks identified for the 226 differentially expressed proteins included cellular movement, assembly and organization (score = 41) and cell morphology (score = 20) (Supplementary Table 7), consistent with the biological processes affected by the overexpression of miR-93 as observed in the previous in vitro experiments.

To confirm the most relevant results of the proteomic analysis, we selected a group of proteins significantly dysregulated (CRMP2, ITGA2, MAD2L1, CDK1, CHMP4B, MAPRE1, and YES1) that are involved in cell cycle, mainly G2/M or cytokinesis, cell adhesion, cell migration, or microtubule organization, and we evaluated their levels in HPDE and PANC-1 models by Western Blot (Supplementary Fig. 6). Consistently, CRMP2, ITGA2, MAD2L1, and CDK1 were more abundant in HPDE-hTR control cells than in HPDE-miR-93 and more expressed in PANC-1 KO-miR-93 than in control PANC-1 as we would expect. CHMP4B, only detected by WB in PANC-1 cells, was also more abundant in the absence of miR-93 than in control cells. Conversely, MAPRE1 levels in HPDE cells disappeared when miR-93 was overexpressed while this protein was hardly detected in PANC-1 cells in general. Finally, YES1 expression was higher in PANC-1 KO-miR-93 compared to control PANC-1 cells, but in contrast to previous proteomic analysis WB did not show a decrease in HPDE cells overexpressing miR-93.

### CRMP-2, YES1, and MAPRE1 are direct targets of miR-93

Taking into account these results and miRNA-target prediction databases, we considered CRMP2 (collapsin response mediator protein, also known as DPYSL2), YES1 (YES proto-oncogene 1 from Src family tyrosine kinase), and MAPRE1 (microtubule associated protein RP/EB family member 1, also known as EB1) as interesting miR-93 target candidates to be further evaluated. They were predicted to be potentially targeted by miR-93 by 8, 9, and 9 databases, respectively, using the miRWalk 2.0 platform, which integrates 12 different target prediction algorithms. Moreover, analysis of 3′UTR sequences of the corresponding mRNAs confirmed the presence of at least 1 binding site for miR-93 (CGUGAA) in all of them.

**Fig. 6:**
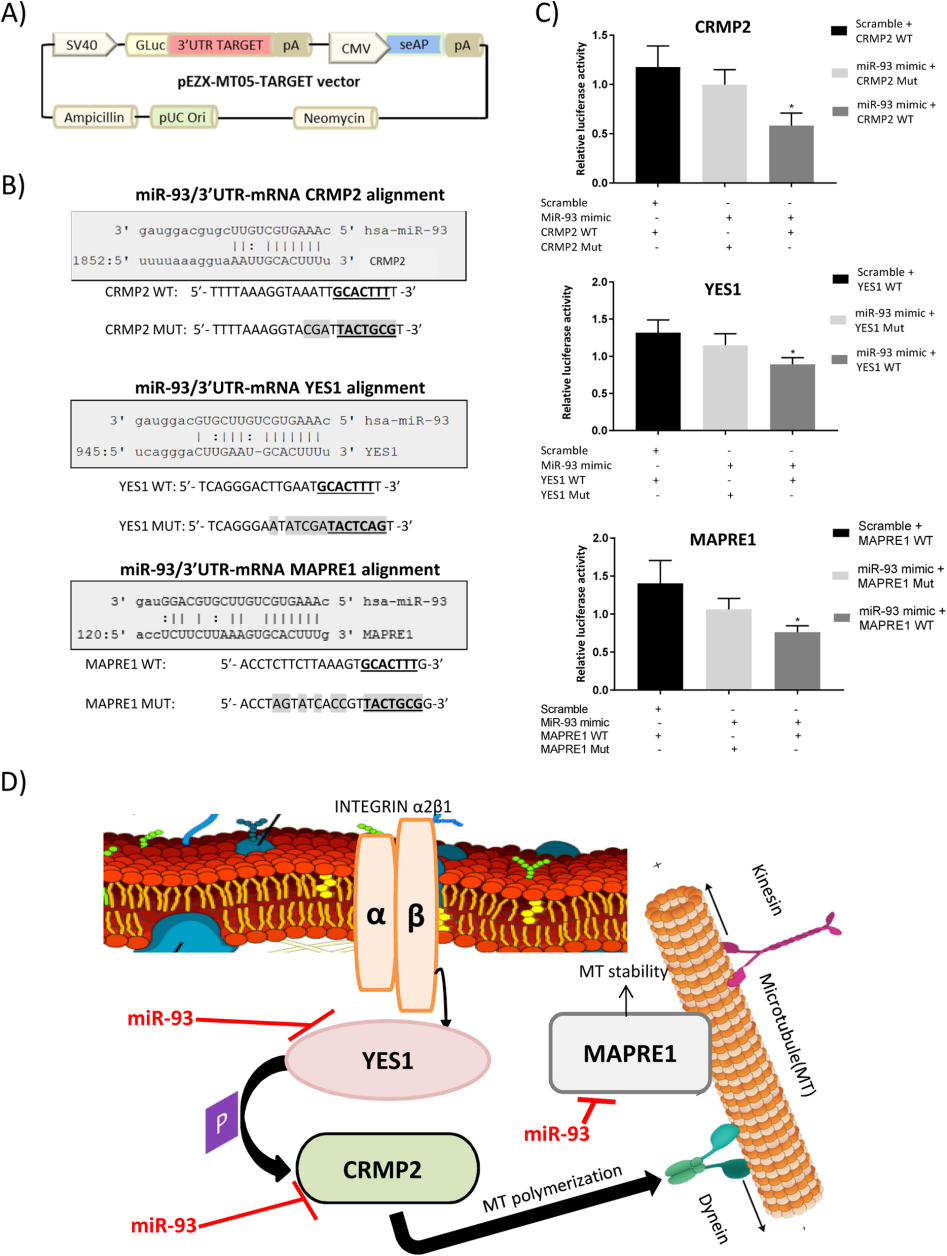
CRMP2, YES1, and MAPRE1 are direct targets of miR-93. **a** Schematic representation of pEZX-MT05 vector. **b** Predicted interactions between miR-93 and CRMP2, YES1, and MAPRE1 mRNA 3′-UTR region. **c** CRMP2, YES1, and MAPRE1 luciferase reporter assays (wild-type and mutated) in HEK293T cells transfected with either miR-93 mimic or scramble; luciferase activity normalized against alkaline phosphatase activity; *n* = 3, error bars = s.d., *p ≤ 0.05. **d** Schematic representation of miR-93 regulation of the proposed targets in PDAC. In this context, miR-93 demonstrates the ability of targeting multiple protein coding genes from the same signaling pathway (microtubule dynamics), at different levels.

In order to examine the direct binding of miR-93 to the potential target genes CRMP2, YES1, or MAPRE1, luciferase reporter assays were performed. A region of the 3′-UTR of CRMP2, YES1, or MAPRE1 mRNA containing the seed sequence of miR-93 was placed downstream of the firefly luciferase gene in a reporter plasmid (Fig. 6a). The predicted interactions between miR-93 and the target sites in the CRMP2, YES1, and MAPRE1 3′-UTR are illustrated in Fig. 6b. The resulting plasmids were transfected into HEK293T cells along with miR-93 mimic or a scramble, and the luciferase activity was evaluated. As a result, luciferase activity was significantly reduced in cells transfected with miR-93 mimic in all cases (Fig. 6c). These results demonstrate that CRMP2, YES1, and MAPRE1 are direct targets of miR-93. Figure 6d depicts a model with the found miR-93 targets all involved in microtubule dynamics at different levels.

## Discussion

In the present study, we have deciphered an oncogenic role of miR-93 in the context of PDAC by modulating constitutively its expression using two different approaches: a loss-of-function approach generated by CRISPR–Cas9 technology to abrogate miR-93 expression in two pancreatic cancer cellular models and a gain-of-function approach generated by retroviral transduction to stably overexpress miR-93 in a normal pancreatic cellular model. We have focused our efforts in this particular miRNA as we previously found it significantly overexpressed in tumor samples from patients with PDAC^17,18^. Moreover, we have seen here that tumor high levels of miR-93 significantly correlate with a worst prognosis of the disease, suggesting an implication of this miRNA in pancreatic carcinogenesis.

MiR-93 involvement in PDAC has been vaguely approached, with only one study reporting that RUNX1, a key transcription factor in haematopoiesis, negatively regulates the expression of miR-93^20^. Although some studies have described oncogenic functions for this miRNA in other cancers^21,22^, other reports have found that miR-93 can also act as a tumor suppressor in other contexts^23,24^, confirming the fact that the expression and function of a miRNA is context-dependent.

To study the molecular function of miR-93 in PDAC, we first depleted miR-93 in two pancreatic cancer cell lines, PANC-1 and MIA PaCa-2, with CRISPR/Cas9 technology and, by doing so, we came across with a strikingly different cellular morphology. Cells with miR-93 depletion were larger in size and multinucleated in most cases, and they divided more slowly than control cells. On the contrary, when we overexpressed miR-93 in the nontumor pancreatic cell line, HPDE, cells acquired a pro-tumorigenic phenotype. These cells had a significantly higher proliferation rate, were able to close a wound gap significantly faster and displayed higher invasion capacity than control HPDE cells. Flow cytometry assays demonstrated that inhibition of miR-93 in PANC-1 cells induced these cells to an arrest at G2/M, which was partially recovered when miR-93 was re-expressed in these cells by transient transfection. Next, we confirmed in vivo the effects observed in vitro. Actually, heterotopic tumor xenografts generated by PANC-1 KO-miR-93 cells had a significantly slower growth than control PANC-1 tumors. At necropsy, these tumors confirmed to be of smaller size, reduced weight, less proliferative according to Ki67 and H&E staining showed cell enlargement. With all these observations, we dare to propose miR-93 as an active molecule of the pancreatic carcinogenesis.

In order to deeply understand the functional activity of miR-93, it is essential to know which main targets or signaling pathways are under the control of its regulation. Since miRNAs mainly act repressing translation of mRNAs, we decided to perform proteomic analysis of our cellular models in which miR-93 expression had been altered. Proteomic results were clearly aligned with the observed behavior in vitro as the list of dysregulated proteins were mainly involved in the regulation of the cell cycle, specifically G2/M phase, and microtubule dynamics. From these results, we could infer an important regulatory role of miR-93 on these molecular pathways.

As we have shown here, miR-93 levels are negatively related to CHMP4B, which is highly increased in PANC-1 KO-miR-93. CHMP4B is a component of the endosomal sorting complex required for transport (ESCRT)-III^25^ that functions in membrane fission events, such as the terminal stages of cytokinesis^26^. Vietri M et al. reported that interference with ESCRT-III functions in anaphase, as it may happen by depleting miR-93, is accompanied by delayed microtubule disassembly, compromised nuclear integrity, and the appearance of DNA damage foci in subsequent interphase^27^.

We have also observed that MAD2L1, a component of the spindle-assembly checkpoint that prevents the onset of anaphase until all chromosomes are properly aligned at the metaphase plate^28^, is altered by the modulation of miR-93. The fact that MAD2L1 expression increases when miR-93 is absent in PANC-1 KO-miR-93 cells, agrees with the observed phenotype of many of these cells arrested at M phase. Conversely, a decrease of MAD2L1 protein levels is observed in HPDE-miR-93 expressing cells, suggesting that these cells may move forward without a proper cell division.

In addition, CDK1, a key molecule in the control of the eukaryotic cell cycle that promotes G2–M transition^29^, is also altered by miR-93 modulation, confirming an important role of this miRNA in this cell cycle phase. More specifically, once chromosomes are condensed and aligned at the metaphase phase, CDK1 activity is switched off by WEE1- and PKMYT1-mediated phosphorylation to allow cytokinesis and it accumulates in mitochondria in G2-arrested cells upon DNA damage^30^, in accordance with what happens in PANC-1 KO-miR-93 cells.

Furthermore, YES1 (YES proto-oncogene 1), along with other members of the Scr protein-tyrosine kinase family, Fyn, and c-Src, is involved in G2/M progression and cytokinesis^31^. Herein, we have found that miR-93 directly targets YES1, reaffirming again the implication of this miRNA in these cellular processes. YES1 levels are increased in both PANC-1 KO-miR-93 and HPDE-miR-93, possibly triggering different effects in two different cellular contexts. It has been reported that YES1 phosphorylates CRMP2 but the biochemical and functional effect of such phosphorylation has not been identified yet^32^.

We have demonstrated that CRMP2 (collapsin response mediator protein, also known as DPYSL2) is also a direct target of miR-93. CRMP2 protein levels decrease upon miR-93 overexpression and increase with its absence. CRMP2 is a key modulator of microtubule dynamics that binds to tubulin heterodimers to promote microtubule assembly^33^, however, the signaling pathways that regulates its interaction with microtubules have remained poorly characterized. Its most widely described role is in relation to neuronal development and polarity, axon growth and guidance but its expression and function in non-neuronal cells is quite unknown^34^. CRMP2 can be phosphorylated by GSK3β, CDK5, FER, and Rho kinase which impairs or reduce its ability to bind to tubulin and to induce microtubule assembly^35–38^.

Likewise, MAPRE1 (microtubule associated protein RP/EB family member 1, also known as EB1), found overexpressed in PANC-1 KO-miR-93 protein extracts and confirmed as a direct target of miR-93, is a microtubule (MT) plus end-tracking protein (+TIPS) that promotes cytoplasmic MT nucleation and elongation^39^, is involved in spindle function by stabilizing microtubules and anchoring them at centrosomes^40^ and suppresses catastrophes^41^. The remodeling of MT, filamentous structures required for cellular processes such as intracellular transport, cell division, and locomotion, depends on MT dynamic instability, spontaneous switching between episodes of growth and shortening^42^. With the absence of miR-93, MAPRE1 levels increase probably inducing a more permanent MT stability provoking defects in mitosis resolution.

On the other hand, miR-93 levels are also negatively related to ITGA2 protein levels. Integrin α2 subunit (ITGA2) together with the β1 integrin subunit (ITGB1), is a heterodimer receptor for matrix molecules (laminin, collagen, fibronectin,…), cell surface (e.g., E-cadherin) and vascular cell adhesion molecules^43^. In this study, the reduction of ITGA2 observed in HPDE-miR-93 expressing cells is consistent with a phenotype transformation to a more invasive state. In accordance, it has been reported that low levels of ITGA2 benefit breast cancer cells to detach the extracellular matrix of primary tumor to facilitate invasion and metastases^44^.

Here, we speculate that miR-93 in the PDAC context is mainly implicated in the regulation of the microtubule dynamics by directly regulating the expression of YES1, CRMP2, and MAPRE1 (Fig. 6d). Thus, an aberrant increase in miR-93 would lead to a reduction in YES1 protein levels; thence, its kinase activity involved in regulation of G2/M progression and cytokinesis, and also on CRMP2, would be impaired. Likewise, CRMP2 protein levels would decrease, impairing its effects on tubulin heterodimers leading to an increase in the MT disassembly rate. In the same way, MAPRE1 protein levels would also decrease thus inducing MT instability. Therefore, disruption of the harmonious transcriptional tuning of the microtubule dynamics signaling pathway at different levels would produce aberrant faster mitosis and subsequent chromosomal instability, agreeing with a pro-oncogenic phenotype. However, understanding the exact mechanism that occurs through these targets in the context of pancreatic cancer goes beyond the scope of this study, and further experiments will be required.

In conclusion, we have demonstrated here that miR-93 exerts oncogenic functions in the pancreatic cancer context. It regulates the pancreatic tumor phenotype especially modulating the expression of critical genes involved in microtubule dynamics and resolution of mitosis and cytokinesis, among others. MiR-93 could be considered as a potential prognostic biomarker for PDAC, and miR-93 itself or its protein targets (CRMP2, MAPRE1, or YES1) have been elucidated as potential new therapeutic targets for this cancer. However, validation studies in other cohorts of patients and further experiments would be required before translating these results into the clinics.

## Supplementary information

Supplementary methods

Supplementary figure and table legends

Supplementary figure 1

Supplementary figure 2

Supplementary figure 3

Supplementary figure 4

Supplementary figure 5

Supplementary figure 6

Supplementary table 1

Supplementary table 2

Supplementary table 3

Supplementary table 4

Supplementary table 5

Supplementary table 6

Supplementary table 7
