# Supplementary material for: MiR-93 is related to poor prognosis in pancreatic cancer and promotes tumor progression by targeting microtubule dynamics
Source: Oncogenesis. 2020 May 4;9(5):43. doi: 10.1038/s41389-020-0227-y (PMC7198506; doi:10.1038/s41389-020-0227-y)
Supplement: Supplementary file 1 — Supplementary methods [file 41389_2020_227_MOESM1_ESM.docx]

**SUPPLEMENTARY MATERIALS AND METHODS**

### Retrovirus production and HPDE infection for stable overexpression of miRNA

We used the retroviral expression vector miRVec-93 (pMSCV-Blasticidin plasmid) obtained from the miR-Lib microRNA library (Source Bioscience, Nottingham, UK). MiRVec-93 originally contained the whole sequence of pre-miR-93 plus miR-25 and half the sequence of miR-106b under the control of a CMV promoter. From this vector we sub-cloned miR-93 genomic region. The region of 327 bp containing miR-93 was amplified by high fidelity PCR (Thermo Fisher Scientific) and cloned into an empty miRVec vector using the restriction sites of BamH1 and EcoR1. Confirmation of the cloning was performed by Sanger sequencing (Beckman Coulter Genomics, Takeley, Essex, United Kingdom). As a plasmid control, we used miRVec-hTR that contains the sequence for the RNA subunit of the telomerase enzyme and is similar in size to the sequence of miRNAs.

MiRVecs were transfected into HEK293Φ cells, by using the CalPhos mammalian transfection kit (Clontech Laboratories, Takara Bio Company Inc., Mountain View, CA, USA*),* in order to generate retroviral particles. Virus-containing HEK293Φ supernatants were collected at 48h post-transfection, filtered and used to transduce HPDE cells to stably express miR-93 or hTR. Twenty-four hours post-infection, HPDE cells were treated with 18 μg/mL blasticidin, for a few days to select for transduced cells. Next, serial dilution was carried out to generate individual clones of HPDE-miR-93 expressing cells.

### CRISPR/Cas9 targeting of miR-93 in PANC-1 and MIA PaCa-2 cells

*gRNA design:* the gRNA of miR-93 was designed using the “CRISPR design tool” from Feng Zhang Lab (<http://crispr.mit.edu/>). We chose a PAM sequence (AGG) in the genomic pre-miR-93 region and selected a 20-bp sequence upstream as the targeting sequence (5’- CTCCAAAGTGCTGTTCGTGC-3’). Oligonucleotides of the indicated sequence were purchased from IDT (Leuven, BE), annealed and cloned into the plentiCRISPRv2 vector following Lentiviral CRISPR Tool box instructions from Zhang Lab deposited to Addgene.

*Verification of gRNA-mediated genome cleavage:* HEK293T cells were transfected with the plentiCRISPRv2 containing miR-93 gRNA by using the CalPhos mammalian transfection kit (Clontech). Cells were treated with 4 μg/ml puromycin for one week. Next, genomic DNA from transfected and wild-type cells was isolated and submitted to PCR amplification of a 705bp fragment that encompasses miR-93 region using the following primers: Fwd: 5’-tgagggagaccagacccttt-3’ and Rev: 5’- ttctgcttccccatgaacct -3’. PCR fragments were subjected to SURVEYOR nuclease assay (Integrated DNA Technologies, Inc., Coralville, Iowa, USA) and resolved on 1.5% agarose gel. Mutations were confirmed by DNA sequencing.

*Generation of miR-93-deleted PANC-1 and MIA PaCa-2 cells:* Lentiviral particles were generated by transfection of vectors plentiCRISPRv2miR-93gRNA or plentiCRISPRv2-Control, pVSV-G and pCMVΔ8.91 into HEK293T by using the CalPhos mammalian transfection kit. At 48h the viral supernatants were collected, filtered and used to transduce PANC-1 and MIA PaCa-2 cells. Three days after transduction, cells were selected in 8 μg/ml puromycin for one week. Next, limiting dilution was carried out to generate individual clones of infected gRNA93 cells and three weeks later several clones were analyzed for DNA mutation and miR-93 expression.

### Senescence assay

PANC-1 KO-miR-93 and control PANC-1 cells were seeded at 4x10^4^ cells in a six well plate 48h prior to staining. We used the *Senescence β-Galactosidase Staining Kit* (Cell Signaling Technology Inc., MA, USA). Cells were first fixed for 10-15 min, β-Galactosidase staining solution was added to each well and the cell plate was sealed and incubated over-night at 37ºC according to the manufacturer’s instructions. Cells were checked under a microscope for the development of blue color, and blue-stained cells (senescent cells) were counted.

### Binucleate assay

PANC-1 KO-miR-93 cells and control PANC-1 cells were trypsinized, centrifuged and treated with hypotonic solution (KCl 0.075M at 4ºC). Cells were re-centrifuged and ﬁxed with ﬁxative (methanol:glacial acetic acid, 3:1) + 3 drops of formaldehid 37%. Fixation was repeated twice with methanol:glacial acetic acid (3:1). Microscope slides were prepared in triplicate by dropping cell samples, incubating them 5min with 2x SSC, dehydrating with 70, 90 and 100% EtOH for 1 min each, airdrying and staining with Giemsa solution (pH=6.8):PBS, 1:9 for 15 min. They were ﬁnally washed in distilled water and dried at room temperature.

### Transient transfection of miR-93 mimic

Control PANC-1 and PANC-1 KO-miR-93 cells were seeded at 6•10^4^ in a 6-well plate. Immediatelly, Lipofectamine LTX (Thermo Scientific, San Jose, CA, USA) transfection mix containing 2500 ng of miR-93 mimic (Ambion, Austin, TX, USA) or miRNA negative control (Ambion) was added to the wells according to manufacturer’s instructions. After 48h, miR-93 overexpression was confirmed by qRT-PCR. Cells were then harvested for further analysis.

### Analysis of cell cycle by flow cytometry

Control PANC-1 and PANC-1 KO-miR-93 cells were seeded at 6·10^5^ in a 10cm^2^ dish. 24h later, supernatant was harvested, cells were digested with trypsin and collected along with the supernatant by centrifugation and washed once with PBS. Cells were resuspended with PBS and fixed by adding drops wise while vortexing, ice-cold 70% ethanol. Cells were then incubated over-night at 4ºC, centrifuged to discard ethanol and washed twice in cold PBS. Fixed cells were treated with RNase A (20μg; Sigma-Aldrich, MO, USA) for 30min at room temperature (RT) and stained with propidium iodide (10μg/ml Sigma-Aldrich) for 10min, RT in the dark. Cell cycle was measured by BD FACSCantoII flow cytometer (Becton Dickinson-BD-San Jose, CA, USA) (488 -585/42-556LP Channel) using FACSDiva 6.1.2 software (BD) and counted with at least 10.000 events. Data analysis was performed using FACSDiva 6.1.2 software.

**Time-lapse live-cell imaging**

PANC-1 control (n=3 wells) and PANC-1 KO-miR-93 (n=5 wells) cells were plated in a µ-Slide 8 Well Chambered Coverslip for Cell Imaging at a confluency of 10^3^ cells per well. Time-lapse transmitted light experiments were carried out using a Leica TCS SP5 laser-scanning confocal spectral microscope (Leica Microsystems Heidelberg, Manheim, Germany) with Argon and HeNe lasers attached to a Leica DMI6000 inverted automated microscope equipped with an incubation system with temperature and CO_2_ control and precise Diode based Autofocus Control. For visualization of dividing cells, Differential Interference Contrast images were acquired using a PL APO CS 20 x objective lens, Numerical Aperture 0.7, and 561 nm laser line. 4 fields were acquired at 3 different heights from each well in order to keep focus of detaching cells due to division. Images were acquired at 3 min intervals for 15 hours in a 10242 x 1024 format and zoom 1. All experiments were performed at 37 ºC and 5% CO2.

### In vivo study

To induce tumor xenografts, 2·10^6^ PANC-1 KO-miR-93 clone1, clone 2 or control PANC-1 cells, embedded in a matrix 1:1 of Matrigel (Corning), were inoculated subcutaneously into each flank of 6-week-old male athymic nude Foxn 1nu nu/nu mice (Envigo, Huntingdon, Cambridgeshire, United Kingdom). Mice were randomly allocated into experimental groups. Total number of injected mice was 21, of which 7 were injected with PANC-1 KO-miR-93 clone1 (n=14 tumors), 7 with PANC-1 KO-miR-93 clone 2 (n=14 tumors) and 7 with control PANC-1 cells (n=14 tumors). Animals were maintained under specific pathogen-free conditions at Scientific and Technological Centre of University of Barcelona and examined every two days to assess general health conditions and evaluate tumor growth. Eight days post-injection, tumor masses were detectable by palpation and tumor size was measured with a manual caliper. Tumor volume was calculated according to the following equation: Volume=$\frac{\pi}{6}\times D\times d^{2}$where *D* is the larger diameter and *d* is the smaller diameter. Mice were euthanized 7 weeks post-inoculation and tumor specimens were excised and weighed. Tumors were fixed in 4% paraformaldehyde for 24h and then processed for haematoxylin and eosin staining. For Ki67 staining (1/2500 diluted antibody; ab92742, Abcam, Cambridge, UK), antigen retrieval by citrate tampon for 20 min followed by Bond Polymer Refine Detection was performed. Investigators were blinded to the group allocation during the experiment and when assessing the outcome. Animal procedures met the guidelines of European Community Directive 86/609/EEC and were approved by the Local Ethical Committee.

### Protein sample preparation for proteomic analysis

Whole-cell protein extracts were obtained with RIPA buffer (without SDS and NP-40 <1%), 1 protease inhibitor cocktail tablet (*Complete Mini;* Roche Diagnostics, Basel, Switzerland) and 1 *PhosStop* tablet (Roche) following standard protocols. Protein samples were sent to the Proteomics Unit at UPF/CRG in Barcelona for further processing and proteomic analysis. 30µg of each sample was precipitated by the addition of six volumes of acetone (overnight, 4ºC). Precipitated proteins were then dissolved in 6M Urea plus 200mM NH_4_HCO_._ Samples were reduced with dithiothreitol (90nmols, 1h, 37°C) and alkylated in the dark with iodoacetamide (180nmol, 30min, 25ºC). The resulting protein extract was first diluted 1/3 with 200mM NH_4_HCO_3_ and digested with 3µg Lys-C (Wako Chemicals, Richmond, VA) overnight at 37ºC and then diluted 1/2 and digested with 3µg of trypsin for 8h at 37˚C. Finally, the peptide mix was acidified with formic acid and desalted with a MicroSpin C18 column (The Nest Group, Inc) prior to LC-MS/MS analysis.

### Chromatographic and mass spectrometric analysis

The peptide mixes were analysed using an Orbitrap Fusion Lumos mass spectrometer (Thermo Scientific, San Jose, CA, USA) coupled to an Easy-LC (Thermo Scientific (Proxeon), Odense, Denmark). Peptides were loaded directly onto the analytical column at a flow rate of 1.5-2μl/min using a wash-volume of 4 times the injection volume and were separated by reversed-phase chromatography using a 50cm column with an inner diameter of 75μm, packed with 2μm C18 particles spectrometer (Thermo Scientific, San Jose, CA, USA). Chromatographic gradients started at 95% buffer A and 5% buffer B with a flow rate of 300 nl/min and gradually increased to 22% buffer B in 79 min and then to 35% buffer B in 11 min. After each analysis, the column was washed for 10 min with 5% buffer A and 95% buffer B. Buffer A: 0.1% formic acid in water. Buffer B: 0.1% formic acid in acetonitrile.

The mass spectrometer was operated in DDA mode and full MS scans with 1 micro scans at resolution of 120.000 were used over a mass range of m/z 350-1500 with detection in the Orbitrap. Auto gain control (AGC) was set to 2E5 and dynamic exclusion to 60 seconds. In each cycle of DDA analysis, following each survey scan Top Speed ions with charged 2 to 7 above a threshold ion count of 1e4 were selected for fragmentation at normalized collision energy of 28%. Fragment ion spectra produced via high-energy collision dissociation (HCD) were acquired in the Ion Trap, AGC was set to 3e4, isolation window of 1.6 m/zand maximum injection time of 40ms was used. All data were acquired with Xcalibur software v3.0.63.

### Protein Data Analysis

Proteome Discoverer software suite (v2.0, Thermo Fisher Scientific) and the Mascot search engine (v2.5, Matrix Science) were used for peptide identification and quantification. Samples were searched against a SwissProt database containing entries corresponding to Human (version, April 2016) a list of common contaminants and all the corresponding decoy entries. Trypsin was chosen as enzyme and a maximum of three miscleavages were allowed. Carbamidomethylation (C) was set as a fixed modification, whereas oxidation (M) and acetylation (N-terminal) were used as variable modifications. Searches were performed using a peptide tolerance of 7 ppm, a product ion tolerance of 0.5 Da. Resulting data files were filtered for FDR < 5 %. Average peak area of the 3 top unique peptides for a given protein have been calculated and normalized by the median. To compare HPDEmiR-93 vs Control and PANC-1 KO-miR-93 vs Control, fold change, pvalue and adjusted pvalue was calculated with Perseus 1.5.5.3.

### Network analysis

The significant differentially expressed proteins between HPDE-hTR and HPDE-miR-93 and between control PANC-1 and PANC-1 KO-miR-93 were characterized by core analysis using the Ingenuity Pathway Analysis (IPA; QIAGEN Inc., <https://www.qiagenbioinformatics.com/products/ingenuitypathway-analysis>) to check the putative enrichment for canonical pathways, disease and biological functions and molecular networks. The IPA networks generation algorithm transformed the protein list into a network set using Global Molecular Network connections and Ingenuity Pathways Knowledge Base. The p-score is defined as the -log_10_(p-value).

### Luciferase Reporter assays

Custom 3’-UTR of candidate target genes MAPRE1 (300bp), YES1 (300bp) and DPYSL2 (300bp) were cloned into the pEZX-MT05 vector (8577bp) downstream of the luciferase gene (GeneCopoeia, Rockville, MD, USA). Reporter constructs containing mutated 3’-UTRs of the same genes and an empty vector (pEZX-MT05) were also purchased from GeneCopoeia. All plasmids were transformed in DH5α competent cells, picked colonies were grown in liquid bacteria (LB)+ampicillin and plasmid DNA were extracted using Maxiprep kit (QIAGEN). A total of 2,2·10^5^ HEK293T cells were seeded per well in 12-well plates. After 24h, 800ng of pEZX-MT05 plasmid (3’-UTR target, mutant 3’-UTR target or control) were co-transfected with 30μM of miR-93 mimic or negative control miRNA mimic (miRVana, Thermo Fisher Scientific) by CalPhos solution (Clontech). After 48h, luciferase activity was assessed with the Secrete-Pair^TM^ luminescence assay kit (GeneCopoeia) following the manufacturer’s protocol. Experiments were done in triplicates.

**Statistical analysis**

For *in vitro* and *in vivo* experiments, data from a minimum of 3 independent experiments are presented as mean ± s.d. Student’s t-test (two-tailed) was used to compare 2 groups and p<0.05 was considered significant. The variance is similar between the groups that are being statistically compared. All statistical analysis and graphs were done using GraphPad Prism version 7.00 for Windows, GraphPad Software, La Jolla California, USA, [www.graphpad.com](http://www.graphpad.com).
