# Supplementary material for: MiR-93 is related to poor prognosis in pancreatic cancer and promotes tumor progression by targeting microtubule dynamics
Source: Oncogenesis. 2020 May 4;9(5):43. doi: 10.1038/s41389-020-0227-y (PMC7198506; doi:10.1038/s41389-020-0227-y)
Supplement: Supplementary file 2 — Supplementary figure and table legends [file 41389_2020_227_MOESM2_ESM.docx]

**supplementary figure and table legends**

**Supplementary figure 1.** **Expression analysis of miR-106b, miR-25 and MCM7 by qRT-PCR.** Endogenous controls: RNU6B for miRNAs and GAPDH for MCM7; error bars: s.d.

**Supplementary figure 2. Characterization of MIA PaCa-2 cells after depletion of miR-93 by CRISPR/Cas9.** A) Schematic representation of the miR-106b-25 cluster and the CRISPR/Cas9 strategy for miR-93 depletion in MIA PaCa-2 cells. B) MiR-93 expression analysis by qRT-PCR in MIA PaCa-2 Control or KO-miR-93 cells, n=2; C) Optical microscope images. Scale bars 10µm, magnitude 10x. D) Doubling time of MIA PACa-2 cells with or without miR-93 in hours, n=3; C1: clone1 and C2: clone2 . E) MTS assay to show the effects of miR-93 depletion on MIA PaCa-2 cell proliferation and viability (n=3). F) Transwell invasion assay of MIA PaCa-2 KO-miR-93 cells compared with MIA PaCa-2 Control cells (n=3). Statistics for number of invading cells per insert, representative images of the analysed conditions magnitude 4x, scale bars 100µm. Error bars=s.d.; *p≤0.05 **p≤0.01; *** p≤ 0.001.

**Supplementary figure 3. Movie.** Representative excerpt from a 15-hour time-lapse showing 8 cell divisions of control PANC-1 cells (n=3 wells, 4 fields at 3 different heights per well). White arrows indicate areas with observed cell divisions.

**Supplementary figure 4.** **Movie.** Representative excerpt from a 15-hour time-lapse showing no cell divisions of PANC-1 KO-miR-93 cells (n=5 wells, 4 fields at 3 different heights per well).

**Supplementary figure 5. Canonical pathways from an IPA core analysis run with the PANC-1 proteomics’ dataset.** The significance of the association between the data set and the pathway was determined based on the p-value, which determines the probability that the association between the data set and the pathway is explained only by chance, and on the ratio value, representing the number of proteins from the data found in each pathway over the total number of proteins in that pathway.

**Supplementary figure 6. Western Blot confirms proteomic analysis results on PANC-1 and HPDE cellular models.** Western Blot against proteins involved in the terminal stages of cytokinesis (CHMP4B, MAD2L1), in G2/M transition (CDK1, YES1), in microtubule dynamics (CRMP2, MAPRE1) and in cell adhesion (ITGA2), in both cellular models lacking (PANC-1) or overexpressing miR-93 (HPDE) (n=3 independent experiments). Normalization against GAPDH or Cyclophilin. C1: clone1 and C2: clone2. Representative images are shown.

**Supplementary table 1.** Clinicopathologic characteristics of PDAC patients included in the study.

**Supplementary table 2.** Differentially expressed proteins in control PANC-1 vs PANC-1 KO-miR-93 from the proteomic analysis. Fold change (control vs KO) ≤ -0.5; p-value ≤ 0.05 and protein detection in at least 2 replicas for each condition (n=3).

**Supplementary table 3.** Up-regulated proteins in PANC-1 KO-miR-93 cells from the proteomic analysis. Only those undetected proteins in control cells and detected in 2 or 3 replicas from KO-miR-93 cells are listed (n=3).

**Supplementary table 4.** Top diseases and top 3 networks revealed by IPA analysis according to the proteomic analysis comparing control versus KO-miR-93 PANC-1 cells.

**Supplementary table 5.** Differentially expressed proteins in control HPDE vs HPDE miR-93 from the proteomic analysis. Fold change _(control hTR vs miR-93)_ ≥ 0.5, p-value ≤ 0.05 and protein detection in at least 2 replicas for each condition (n=3).

**Supplementary table 6.** Down-regulated proteins in HPDE-miR-93 cells from the proteomic analysis. Only those proteins detected in 2 or 3 replicas from control cells and undetected in HPDE-miR-93 cells are listed (n=3).

**Supplementary table 7.** Top diseases and top 3 networks revealed by IPA analysis according to the proteomic analysis comparing control versus overexpressing miR-93 HPDE cells.
