# Supplementary material for: MiR-93 is related to poor prognosis in pancreatic cancer and promotes tumor progression by targeting microtubule dynamics
Source: Oncogenesis. 2020 May 4;9(5):43. doi: 10.1038/s41389-020-0227-y (PMC7198506; doi:10.1038/s41389-020-0227-y)
Supplement: Supplementary file 4 — Supplementary figure 2 [file 41389_2020_227_MOESM4_ESM.pptx]

## Slide 1
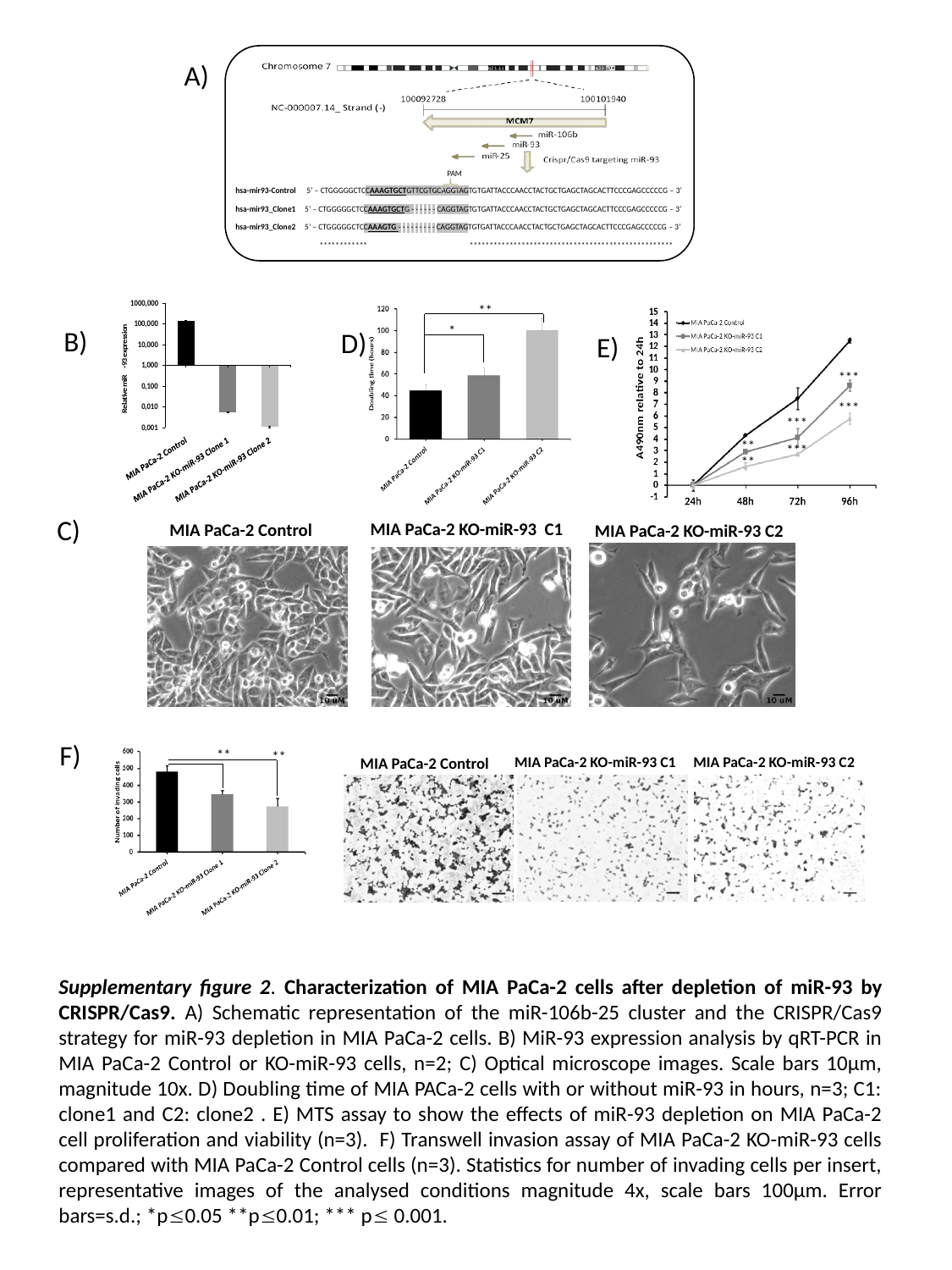

A)
B)
E)
***
***
***
**
***
**
C)
MIA PaCa-2 KO-miR-93 C1
MIA PaCa-2 Control
MIA PaCa-2 KO-miR-93 C2
F)
**
**
MIA PaCa-2 KO-miR-93 C1 Clone 1
MIA PaCa-2 KO-miR-93 C2 Clone 2
MIA PaCa-2 Control
**
*
D)
Supplementary figure 2. Characterization of MIA PaCa-2 cells after depletion of miR-93 by CRISPR/Cas9. A) Schematic representation of the miR-106b-25 cluster and the CRISPR/Cas9 strategy for miR-93 depletion in MIA PaCa-2 cells. B) MiR-93 expression analysis by qRT-PCR in MIA PaCa-2 Control or KO-miR-93 cells, n=2; C) Optical microscope images. Scale bars 10µm, magnitude 10x. D) Doubling time of MIA PACa-2 cells with or without miR-93 in hours, n=3; C1: clone1 and C2: clone2 . E) MTS assay to show the effects of miR-93 depletion on MIA PaCa-2 cell proliferation and viability (n=3). F) Transwell invasion assay of MIA PaCa-2 KO-miR-93 cells compared with MIA PaCa-2 Control cells (n=3). Statistics for number of invading cells per insert, representative images of the analysed conditions magnitude 4x, scale bars 100µm. Error bars=s.d.; *p0.05 **p0.01; *** p≤ 0.001.
