# Supplementary material for: MiR-93 is related to poor prognosis in pancreatic cancer and promotes tumor progression by targeting microtubule dynamics
Source: Oncogenesis. 2020 May 4;9(5):43. doi: 10.1038/s41389-020-0227-y (PMC7198506; doi:10.1038/s41389-020-0227-y)
Supplement: Supplementary file 9 — Supplementary table 1 [file 41389_2020_227_MOESM9_ESM.docx]

**Supplementary table 1**. Clinicopathologic characteristics of PDAC patients included in the study

|  | PDAC (n=83) |
| --- | --- |
| Age. mean (SD) | 64.81 (11.7) |
| Sex. n (%) |  |
| Males | 53 (63.9) |
| Females | 30 (36.1) |
| Tumour stage. n |  |
| I | 19 |
| II | 14 |
| III | 23 |
| IV | 25 |
| Unknown | 2 |
| Resectable. n |  |
| Yes | 33 |
| No | 48 |
| Unknown | 2 |
| Tumour localization. n |  |
| Uncinate process | 1 |
| Head | 57 |
| Body | 15 |
| Tail | 4 |
| Unknown | 6 |
