# Supplementary material for: MiR-93 is related to poor prognosis in pancreatic cancer and promotes tumor progression by targeting microtubule dynamics
Source: Oncogenesis. 2020 May 4;9(5):43. doi: 10.1038/s41389-020-0227-y (PMC7198506; doi:10.1038/s41389-020-0227-y)
Supplement: Supplementary file 10 — Supplementary table 2 [file 41389_2020_227_MOESM10_ESM.docx]

**Supplementary table 2:** Differentially expressed proteins in control PANC-1 vs PANC-1 KO-miR-93 from the proteomic analysis. Fold change _(control vs KO)_ ≤ -0.5; p-value ≤0.05 and protein detection in at least 2 replicas for each condition (n=3).

| **Gene name** | **Fold Change (Control-K0miR93)** | **P value** |  | **Gene name** | **Fold Change (Control-K0milR93)** | **P value** |  | **Gene name** | | **Fold Change (Control-K0miR93)** | **P value** |
| --- | --- | --- | --- | --- | --- | --- | --- | --- | --- | --- | --- |
| ACADVL | -1.01 | 0.000 |  | PYM1 | -0.99 | 0.022 |  | PPT1 | | -0.96 | 0.040 |
| SH3BGRL3 | -2.63 | 0.001 |  | TUBB | -1.71 | 0.022 |  | PAK2 | | -1.52 | 0.041 |
| SCARB2 | -1.11 | 0.001 |  | RRBP1 | -1.61 | 0.022 |  | CCAR1 | | -1.12 | 0.042 |
| AGR2 | -1.23 | 0.002 |  | SEC23IP | -2.6 | 0.023 |  | CCAR2 | | -0.89 | 0.042 |
| MAPRE1 | -2.6 | 0.002 |  | RRAS | -1.75 | 0.024 |  | CTSB | | -0.89 | 0.042 |
| PRCP | -0.82 | 0.003 |  | SEC24C | -1.48 | 0.024 |  | USP5 | | -1.62 | 0.043 |
| VASP | -1.03 | 0.003 |  | YWHAE | -1.45 | 0.024 |  | TUBB3 | | -2.81 | 0.043 |
| CNBP | -1.4 | 0.003 |  | ASNS | -2.03 | 0.025 |  | TUBB4A | | -1.48 | 0.043 |
| MRTO4 | -1.45 | 0.004 |  | FLNB | -1.8 | 0.025 |  | DDX19B | | -1.19 | 0.043 |
| FABP5 | -0.9 | 0.004 |  | STAT3 | -1.54 | 0.025 |  | HSPA6 | | -0.57 | 0.044 |
| CAPZA2 | -1.26 | 0.004 |  | CHP1 | -0.68 | 0.026 |  | AP2B1 | | -1.04 | 0.045 |
| EPS15L1 | -1.65 | 0.005 |  | SPTAN1 | -1.98 | 0.026 |  | SRRM1 | | -0.71 | 0.045 |
| PODXL | -1.97 | 0.005 |  | HMGB1 | -1.27 | 0.026 |  | YWHAB | | -1.41 | 0.046 |
| PPP1R14A | -1.4 | 0.005 |  | TPM3 | -2.07 | 0.026 |  | APOE | | -1.8 | 0.046 |
| LUC7L2 | -1.74 | 0.006 |  | CLPB | -1.5 | 0.026 |  | EIF2S2 | | -0.81 | 0.046 |
| RPRD1B | -0.88 | 0.006 |  | PCNP | -1.12 | 0.027 |  | APEX1 | | -1.27 | 0.046 |
| H1F0 | -1.21 | 0.006 |  | HNRNPUL1 | -1.08 | 0.028 |  | FOXK1 | | -1.16 | 0.046 |
| VCP | -0.87 | 0.007 |  | PTBP1 | -1.16 | 0.028 |  | MOB1B | | -2.6 | 0.047 |
| CDKN2AIP | -1.55 | 0.007 |  | TAX1BP3 | -2.35 | 0.028 |  | AK1 | | -0.91 | 0.047 |
| SMARCC2 | -1.08 | 0.008 |  | ADK | -1.3 | 0.029 |  | MRPL30 | | -1.88 | 0.047 |
| HNRNPK | -0.53 | 0.009 |  | TOLLIP | -1.09 | 0.029 |  | YWHAZ | | -1.61 | 0.047 |
| ACTR1A | -1.96 | 0.009 |  | ATP1B1 | -1.09 | 0.029 |  | MANF | | -0.97 | 0.048 |
| EWSR1 | -0.8 | 0.009 |  | TRAP1 | -1.04 | 0.030 |  | RPSA | | -1.04 | 0.048 |
| FHL2 | -2.05 | 0.009 |  | HMGB2 | -0.85 | 0.030 |  | MSN | | -0.94 | 0.049 |
| NF2 | -2.07 | 0.010 |  | EIF4A3 | -0.55 | 0.030 |  |  |  |  |  |
| GDI2 | -2.44 | 0.011 |  | UGGT1 | -0.71 | 0.030 |  | |  |  |  |
| MCM2 | -2.33 | 0.011 |  | MARCKSL1 | -1.03 | 0.031 |  | |  |  |  |
| MKI67 | -2.99 | 0.013 |  | SNX6 | -0.99 | 0.031 |  | |  |  |  |
| GTF2I | -1.66 | 0.013 |  | DNAJC8 | -1.18 | 0.031 |  | |  |  |  |
| SYPL1 | -1.07 | 0.013 |  | HLA-A | -2.12 | 0.031 |  | |  |  |  |
| RPA1 | -1.66 | 0.014 |  | XRN2 | -1.54 | 0.032 |  | |  |  |  |
| IGF2BP1 | -2.25 | 0.014 |  | CLTC | -0.72 | 0.032 |  | |  |  |  |
| GNG5 | -0.78 | 0.014 |  | CTSZ | -1.24 | 0.032 |  | |  |  |  |
| RCC2 | -1.07 | 0.014 |  | C8orf74 | -1.86 | 0.032 |  | |  |  |  |
| EZR | -1.32 | 0.015 |  | RMDN1 | -0.79 | 0.033 |  | |  |  |  |
| GOPC | -0.91 | 0.015 |  | UFD1L | -1.35 | 0.034 |  | |  |  |  |
| ITGB4 | -0.56 | 0.016 |  | PPP2R1A | -1.32 | 0.034 |  | |  |  |  |
| SMARCE1 | -1.36 | 0.016 |  | HP1BP3 | -1.29 | 0.035 |  | |  |  |  |
| ZNF354A | -0.78 | 0.016 |  | GSTK1 | -0.82 | 0.035 |  | |  |  |  |
| IVD | -0.88 | 0.017 |  | RAB5B | -0.93 | 0.035 |  | |  |  |  |
| EIF6 | -1 | 0.017 |  | GNB1 | -0.5 | 0.036 |  | |  |  |  |
| SDF2L1 | -4.03 | 0.017 |  | FXR2 | -1.07 | 0.036 |  | |  |  |  |
| NSFL1C | -1.8 | 0.018 |  | MCM4 | -2.23 | 0.036 |  | |  |  |  |
| XRCC6 | -0.62 | 0.018 |  | PPP1R10 | -0.98 | 0.036 |  | |  |  |  |
| DAD1 | -0.98 | 0.018 |  | HTATSF1 | -0.61 | 0.037 |  | |  |  |  |
| EEF1A2 | -1.59 | 0.019 |  | TAGLN2 | -2.28 | 0.038 |  | |  |  |  |
| KRT19 | -1.03 | 0.019 |  | G3BP1 | -0.82 | 0.039 |  | |  |  |  |
| PFDN1 | -0.93 | 0.019 |  | TMEM230 | -1.72 | 0.039 |  | |  |  |  |
| CHMP4B | -1.28 | 0.020 |  | SMARCB1 | -1.51 | 0.039 |  | |  |  |  |
| WDR12 | -2.32 | 0.020 |  | HNRNPH2 | -0.74 | 0.040 |  | |  |  |  |
| EMC2 | -1.15 | 0.021 |  | RBBP4 | -0.92 | 0.040 |  | |  |  |  |
| PRMT1 | -1.64 | 0.021 |  | CRIP1 | -0.67 | 0.040 |  | |  |  |  |
| SLC9A3R1 | -1.7 | 0.022 |  | PSME2 | -1.06 | 0.040 |  | |  |  |  |
