# Supplementary material for: MiR-93 is related to poor prognosis in pancreatic cancer and promotes tumor progression by targeting microtubule dynamics
Source: Oncogenesis. 2020 May 4;9(5):43. doi: 10.1038/s41389-020-0227-y (PMC7198506; doi:10.1038/s41389-020-0227-y)
Supplement: Supplementary file 11 — Supplementary table 3 [file 41389_2020_227_MOESM11_ESM.docx]

**Supplementary table 3:** Up-regulated proteins in PANC-1 KO-miR-93 cells from the proteomic analysis. Only those undetected proteins in control cells and detected in 2 or 3 replicas from KO-miR-93 cells are listed (n=3).

|  | **Count** | | | |  | | | | **Count** | | |  | | | | | **Count** | | |
| --- | --- | --- | --- | --- | --- | --- | --- | --- | --- | --- | --- | --- | --- | --- | --- | --- | --- | --- | --- |
| **Gene name** | **Control** | | **KOmiR93** | |  | | **Gene name** | | **Control** | **KOmiR93** | |  | | **Gene name** | | **Control** | | **KOmiR93** |  |
| PURA | 0 | | 2 | |  | | CDK1 | | 0 | 2 | |  | | CHMP2A | | 0 | | 2 |  |
| POLR1D | 0 | | 2 | |  | | NRBP1 | | 0 | 2 | |  | | ADGRA2 | | 0 | | 2 |  |
| PPP2R1B | 0 | | 2 | |  | | SMARCC1 | | 0 | 2 | |  | | UBQLN4 | | 0 | | 2 |  |
| STRN | 0 | | 2 | |  | | CD46 | | 0 | 2 | |  | | DUSP27 | | 0 | | 2 |  |
| PRCC | 0 | | 2 | |  | | DCDC1 | | 0 | 2 | |  | | RNF123 | | 0 | | 2 |  |
| NUP160 | 0 | | 2 | |  | | TBCE | | 0 | 2 | |  | | ADIRF | | 0 | | 2 |  |
| KIAA0754 | 0 | | 2 | |  | | C11orf68 | | 0 | 2 | |  | | JTB | | 0 | | 2 |  |
| GHDC | 0 | | 2 | |  | | FAM210A | | 0 | 2 | |  | | PGP | | 0 | | 2 |  |
| STEAP3 | 0 | | 2 | |  | | PTK2B | | 0 | 2 | |  | | RBM34 | | 0 | | 2 |  |
| ITK | 0 | | 2 | |  | | PABPC4L | | 0 | 2 | |  | | MTA2 | | 0 | | 2 |  |
| DOCK8 | 0 | | 2 | |  | | KRT76 | | 0 | 2 | |  | | DNAH7 | | 0 | | 2 |  |
| CARM1 | 0 | | 2 | |  | | NBAS | | 0 | 2 | |  | | FKBP15 | | 0 | | 2 |  |
| C10orf35 | 0 | | 2 | |  | | NECAP2 | | 0 | 2 | |  | | MAPK3 | | 0 | | 2 |  |
| POP4 | 0 | | 2 | |  | | UBQLN2 | | 0 | 2 | |  | | DPM1 | | 0 | | 2 |  |
| MAD2L1 | 0 | | 2 | |  | | ANO10 | | 0 | 2 | |  | | PIWIL4 | | 0 | | 2 |  |
| NEU1 | 0 | | 2 | |  | | STK36 | | 0 | 2 | |  | | SPNS1 | | 0 | | 2 |  |
| CREB1 | 0 | | 2 | |  | | CYB5R2 | | 0 | 2 | |  | | ALPPL2 | | 0 | | 2 |  |
| DNAJC2 | 0 | | 2 | |  | | BRIX1 | | 0 | 2 | |  | | TRMT6 | | 0 | | 2 |  |
| NCBP2 | 0 | | 2 | |  | | SP3 | | 0 | 2 | |  | | SLC12A2 | | 0 | | 2 |  |
| AXL | 0 | | 2 | |  | | ATL2 | | 0 | 2 | |  | | KIF1BP | | 0 | | 2 |  |
| SPRYD7 | 0 | | 2 | |  | | GID8 | | 0 | 2 | |  | | NIF3L1 | | 0 | | 2 |  |
| UBE3A | 0 | | 2 | |  | | ARFGAP1 | | 0 | 2 | |  | | BAK1 | | 0 | | 2 |  |
| ZNF30 | 0 | | 2 | |  | | LINC00523 | | 0 | 2 | |  | | CPSF3 | | 0 | | 2 |  |
| CYP20A1 | 0 | | 2 | |  | | ARPP21 | | 0 | 2 | |  | | NUBP1 | | 0 | | 2 |  |
| EIF2B3 | 0 | | 2 | |  | | SDCBP | | 0 | 2 | |  | | CCDC12 | | 0 | | 2 |  |
| NOL6 | 0 | | 2 | |  | | PPP2R2D | | 0 | 2 | |  | | RPF2 | | 0 | | 2 |  |
| GATAD2B | 0 | | 2 | |  | | SMARCD2 | | 0 | 2 | |  | | DDX50 | | 0 | | 2 |  |
| POGLUT1 | 0 | | 2 | |  | | ZMYND8 | | 0 | 2 | |  | | GATM | | 0 | | 2 |  |
| ENPP1 | 0 | | 2 | |  | | BET1 | | 0 | 2 | |  | | MBNL1 | | 0 | | 2 |  |
| EBNA1BP2 | 0 | | 2 | |  | | DCPS | | 0 | 2 | |  | | CEPT1 | | 0 | | 2 |  |
| SYN2 | 0 | | 2 | |  | | NIP7 | | 0 | 2 | |  | | MRPL57 | | 0 | | 2 |  |
| DIS3 | 0 | | 2 | |  | | OSTF1 | | 0 | 2 | |  | | AKAP1 | | 0 | | 2 |  |
| MDC1 | 0 | | 2 | |  | | HGS | | 0 | 2 | |  | | RTF1 | | 0 | | 2 |  |
| TMEM115 | 0 | | 2 | |  | | PPME1 | | 0 | 2 | |  | | MSH6 | | 0 | | 2 |  |
| NUBP2 | 0 | | 2 | |  | | FZD7 | | 0 | 2 | |  | | PPAN | | 0 | | 2 |  |
| RIPK2 | 0 | | 2 | |  | | TGM2 | | 0 | 2 | |  | | NELFA | | 0 | | 2 |  |
| PITPNA | 0 | | 2 | |  | | DSCAM | | 0 | 2 | |  | | IWS1 | | 0 | | 2 |  |
| MPP6 | 0 | | 2 | |  | | SERPINB6 | | 0 | 2 | |  | | KIF4A | | 0 | | 2 |  |
| GGCX | 0 | | 2 | |  | | BSDC1 | | 0 | 2 | |  | | CCDC50 | | 0 | | 2 |  |
| ANAPC7 | 0 | | 2 | |  | | WDHD1 | | 0 | 2 | |  | | UXS1 | | 0 | | 2 |  |
| DDX52 | 0 | | 2 | |  | | MAG | | 0 | 2 | |  | | CLN6 | | 0 | | 2 |  |
| NELFE | 0 | | 2 | |  | | TIPRL | | 0 | 2 | |  | | RANBP3 | | 0 | | 2 |  |
| RHPN2 | 0 | | 2 | |  | | ARID4B | | 0 | 2 | |  | | PTPRJ | | 0 | | 2 |  |
| RRS1 | 0 | | 2 | |  | | DSP | | 0 | 2 | |  | | ATP2B4 | | 0 | | 2 |  |
| LUZP1 | 0 | | 2 | |  | | VPS18 | | 0 | 2 | |  | | MLF2 | | 0 | | 2 |  |
| AP3D1 | 0 | | 2 | |  | | SAP30 | | 0 | 2 | |  | | OCRL | | 0 | | 2 |  |
| NOMO2 | 0 | | 2 | |  | | TMOD3 | | 0 | 2 | |  | | SCRIB | | 0 | | 2 |  |
| SMCO2 | 0 | | 2 | |  | | NARS2 | | 0 | 2 | |  | | IFITM3 | | 0 | | 2 |  |
| LAMB1 | 0 | | 2 | |  | | XPO5 | | 0 | 2 | |  | | EDC4 | | 0 | | 2 |  |
| NECTIN2 | 0 | | 2 | |  | | BID | | 0 | 2 | |  | | IL22RA2 | | 0 | | 2 |  |
| CRK | 0 | | 2 | |  | | THUMPD1 | | 0 | 2 | |  | | NBN | | 0 | | 2 |  |
| GPSM1 | 0 | | 2 | |  | | ZNF207 | | 0 | 2 | |  | | RFC2 | | 0 | | 2 |  |
| SMARCD1 | 0 | | 2 | |  | | CCDC6 | | 0 | 2 | |  | | CLCN1 | | 0 | | 2 |  |
| YES1 | 0 | | 2 | |  | | RAB3GAP2 | | 0 | 2 | |  | | CKMT1A | | 0 | | 2 |  |
| MSH2 | 0 | | 2 | |  | | CDC42SE2 | | 0 | 2 | |  | | DIDO1 | | 0 | | 2 |  |
|  | | **Count** | | | |  | |  | | | **Count** | | | |  |  |  |  |  |
| **Gene name** | | **Control** | | **KOmiR93** | |  | | **Gene name** | | | **Control** | | **KOmiR93** | |  |  |  |  |  |
| RPAP3 | | 0 | | 2 | |  | | UBQLN1 | | | 0 | | 3 | |  |  |  |  |  |
| MYBBP1A | | 0 | | 2 | |  | | NXN | | | 0 | | 3 | |  |  |  |  |  |
| PIEZO2 | | 0 | | 2 | |  | | SEC23B | | | 0 | | 3 | |  |  |  |  |  |
| TTC38 | | 0 | | 2 | |  | | BLVRB | | | 0 | | 3 | |  |  |  |  |  |
| PKP2 | | 0 | | 2 | |  | | PPIL4 | | | 0 | | 3 | |  |  |  |  |  |
| COQ6 | | 0 | | 2 | |  | | ASH2L | | | 0 | | 3 | |  |  |  |  |  |
| ELMO2 | | 0 | | 2 | |  | | SVIL | | | 0 | | 3 | |  |  |  |  |  |
| SENP3 | | 0 | | 2 | |  | | LYPLA1 | | | 0 | | 3 | |  |  |  |  |  |
| MLH1 | | 0 | | 2 | |  | | LARP7 | | | 0 | | 3 | |  |  |  |  |  |
| ILK | | 0 | | 2 | |  | | HAT1 | | | 0 | | 3 | |  |  |  |  |  |
| PSMB8 | | 0 | | 2 | |  | | OSBP | | | 0 | | 3 | |  |  |  |  |  |
| SLC4A2 | | 0 | | 2 | |  | | GYG1 | | | 0 | | 3 | |  |  |  |  |  |
| WDR82 | | 0 | | 2 | |  | | AP2A1 | | | 0 | | 3 | |  |  |  |  |  |
| ATPAF1 | | 0 | | 2 | |  | | EXOSC8 | | | 0 | | 3 | |  |  |  |  |  |
| MCMBP | | 0 | | 2 | |  | | ACSF3 | | | 0 | | 3 | |  |  |  |  |  |
| PRUNE2 | | 0 | | 2 | |  | | YBX2 | | | 0 | | 3 | |  |  |  |  |  |
| CAP2 | | 0 | | 2 | |  | | PPP4R3A | | | 0 | | 3 | |  |  |  |  |  |
| NCAPH | | 0 | | 2 | |  | | CLN5 | | | 0 | | 3 | |  |  |  |  |  |
| ASUN | | 0 | | 2 | |  | | DFFA | | | 0 | | 3 | |  |  |  |  |  |
| NAA25 1 | | 0 | | 2 | |  | | LRWD1 | | | 0 | | 3 | |  |  |  |  |  |
| FAHD2B | | 0 | | 2 | |  | | DIEXF | | | 0 | | 3 | |  |  |  |  |  |
| UFC1 | | 0 | | 2 | |  | | CKB | | | 0 | | 3 | |  |  |  |  |  |
| GMPR2 | | 0 | | 2 | |  | | CSNK2A2 | | | 0 | | 3 | |  |  |  |  |  |
| RHEB | | 0 | | 2 | |  | | MBNL2 | | | 0 | | 3 | |  |  |  |  |  |
| DNPEP | | 0 | | 2 | |  | | CCDC142 | | | 0 | | 3 | |  |  |  |  |  |
| SMC2 | | 0 | | 2 | |  | | DERL2 | | | 0 | | 3 | |  |  |  |  |  |
| SPAG7 | | 0 | | 2 | |  | | PTP4A1 | | | 0 | | 3 | |  |  |  |  |  |
| HMBS | | 0 | | 2 | |  | | ATXN2 | | | 0 | | 3 | |  |  |  |  |  |
| ARPP19 | | 0 | | 2 | |  | | PHC2 | | | 0 | | 3 | |  |  |  |  |  |
| STAM2 | | 0 | | 2 | |  | | C8orf82 | | | 0 | | 3 | |  |  |  |  |  |
| RPL26L1 | | 0 | | 2 | |  | | POLR2B | | | 0 | | 3 | |  |  |  |  |  |
| DYNC1LI1 | | 0 | | 2 | |  | | RGS10 | | | 0 | | 3 | |  |  |  |  |  |
| SRPK1 | | 0 | | 2 | |  | | LIPA | | | 0 | | 3 | |  |  |  |  |  |
| ACBD3 | | 0 | | 2 | |  | | VPS28 | | | 0 | | 3 | |  |  |  |  |  |
| PGM5 | | 0 | | 2 | |  | | SNX5 | | | 0 | | 3 | |  |  |  |  |  |
| PABPC1L2A | | 0 | | 2 | |  | | TRMT61A | | | 0 | | 3 | |  |  |  |  |  |
| MED12L | | 0 | | 2 | |  | | CLUH | | | 0 | | 3 | |  |  |  |  |  |
| IDI1 | | 0 | | 2 | |  | | PRDX2 | | | 0 | | 3 | |  |  |  |  |  |
| SCAF4 | | 0 | | 2 | |  | | AP2S1 | | | 0 | | 3 | |  |  |  |  |  |
| METTL7A | | 0 | | 2 | |  | | ZC3H4 | | | 0 | | 3 | |  |  |  |  |  |
| DCTN3 | | 0 | | 2 | |  | |  |  |  |  |  |  |  |  |  |  |  |  |
| TOR3A | | 0 | | 2 | |  | |  |  |  |  |  |  |  |  |  |  |  |  |
| PPP1R18 | | 0 | | 2 | |  | |  |  |  |  |  |  |  |  |  |  |  |  |
| RAB8B | | 0 | | 2 | |  | |  |  |  |  |  |  |  |  |  |  |  |  |
| GGCT | | 0 | | 2 | |  | |  |  |  |  |  |  |  |  |  |  |  |  |
| WASHC2A | | 0 | | 2 | |  | |  |  |  |  |  |  |  |  |  |  |  |  |
| ABR | | 0 | | 3 | |  | |  |  |  |  |  |  |  |  |  |  |  |  |
| SSU72 | | 0 | | 3 | |  | |  |  |  |  |  |  |  |  |  |  |  |  |
| SMARCA5 | | 0 | | 3 | |  | |  |  |  |  |  |  |  |  |  |  |  |  |
