# Supplementary material for: MiR-93 is related to poor prognosis in pancreatic cancer and promotes tumor progression by targeting microtubule dynamics
Source: Oncogenesis. 2020 May 4;9(5):43. doi: 10.1038/s41389-020-0227-y (PMC7198506; doi:10.1038/s41389-020-0227-y)
Supplement: Supplementary file 12 — Supplementary table 4 [file 41389_2020_227_MOESM12_ESM.docx]

**Supplementary table 4.** Top diseases and top 3 networks revealed by IPA analysis according to the proteomic analysis comparing control versus KO-miR-93 PANC-1 cells.

| Top Diseases and disorders | p-value range | No. of proteins |
| --- | --- | --- |
| Infectious diseases | 1,91E-02 – 9,16E-06 | 70 |
| Cancer | 1,91E-02 – 1,27E-05 | 357 |
| Organismal injury and abnormalities | 1,91E-02 – 1,27E-05 | 357 |

| Top Networks | Score | Focus molecules |
| --- | --- | --- |
| 1. Cellular compromise, cell cycle, cellular assembly and organization | 43 | 30 |
| 1. Cellular development, cellular growth and proliferation, cell morphology | 39 | 28 |
| 1. Cell morphology, cellular assembly and organization, cellular compromise | 39 | 28 |
