# Supplementary material for: MiR-93 is related to poor prognosis in pancreatic cancer and promotes tumor progression by targeting microtubule dynamics
Source: Oncogenesis. 2020 May 4;9(5):43. doi: 10.1038/s41389-020-0227-y (PMC7198506; doi:10.1038/s41389-020-0227-y)
Supplement: Supplementary file 13 — Supplementary table 5 [file 41389_2020_227_MOESM13_ESM.docx]

**Supplementary table 5:** Differentially expressed proteins in control HPDE vs HPDE miR-93 from the proteomic analysis. Fold change _(control hTR vs miR-93)_ ≥0.5, p-value≤0.05 and protein detection in at least 2 replicas for each condition (n=3).

| **Gene name** | **Fold Change (Controlvs miR93)** | **P value** |
| --- | --- | --- |
| COL4A3BP | 0.57 | 0.000 |
| HMGA2 | 1.44 | 0.000 |
| LAMB3 | 1.31 | 0.000 |
| IKBIP | 4.15 | 0.001 |
| MSH2 | 1.50 | 0.001 |
| OCIAD2 | 0.95 | 0.001 |
| EML4 | 1.46 | 0.001 |
| PYGM | 0.95 | 0.001 |
| UBE2L6 | 1.57 | 0.001 |
| PFKP | 0.60 | 0.001 |
| SAMHD1 | 0.64 | 0.002 |
| TJP2 | 0.79 | 0.002 |
| CYGB | 0.65 | 0.002 |
| AGRN | 0.80 | 0.003 |
| ANXA6 | 1.73 | 0.003 |
| ERAP1 | 0.71 | 0.004 |
| CDK5RAP3 | 1.32 | 0.005 |
| LAMC2 | 0.82 | 0.005 |
| **CRMP2** | 1.37 | 0.005 |
| CMBL | 0.51 | 0.005 |
| DNAJC17 | 2.18 | 0.006 |
| CORO1A | 0.71 | 0.006 |
| PYCARD | 0.71 | 0.006 |
| CARHSP1 | 0.81 | 0.006 |
| UROD | 0.52 | 0.007 |
| AKAP12 | 1.27 | 0.008 |
| RPL27A | 0.83 | 0.009 |
| RUFY1 | 0.61 | 0.009 |
| BRK1 | 0.66 | 0.012 |
| LSR | 0.90 | 0.012 |
| GRN | 1.32 | 0.012 |
| PARP14 | 1.04 | 0.012 |
| IFIT5 | 0.57 | 0.012 |
| EFHD2 | 1.00 | 0.013 |
| HLA-B | 1.26 | 0.013 |
| PLOD3 | 0.98 | 0.015 |
| TOR1AIP1 | 0.50 | 0.016 |
| SERPINB8 | 0.52 | 0.020 |
| HARS2 | 1.47 | 0.021 |
| SLC25A1 | 0.65 | 0.021 |
| ITGA2 | 0.92 | 0.021 |
| AARS2 | 0.63 | 0.023 |
| ALDH1A3 | 0.63 | 0.023 |
| NAGK | 1.09 | 0.024 |
| ASL | 0.57 | 0.024 |
| MINK1 | 0.70 | 0.024 |
| TAB1 | 1.68 | 0.025 |
| REEP5 | 0.60 | 0.026 |
| IFIT3 | 0.74 | 0.026 |
| FN3KRP | 0.58 | 0.027 |
| NEDD1 | 0.57 | 0.028 |
| SEC61B | 0.70 | 0.028 |
| CETN2 | 0.56 | 0.030 |
| PTGS1 | 1.28 | 0.030 |

| **Gene name** | **Fold change (Controlvs miR93)** | **P value** |
| --- | --- | --- |
| ALCAM | 0.78 | 0.030 |
| TAPBP | 0.70 | 0.030 |
| LNPEP | 0.55 | 0.030 |
| PLOD2 | 1.11 | 0.031 |
| B2M | 0.87 | 0.031 |
| TWF2 | 0.55 | 0.032 |
| OCRL | 4.22 | 0.033 |
| NIN | 0.89 | 0.033 |
| TAP1 | 1.16 | 0.034 |
| AP2A1 | 0.53 | 0.035 |
| UTP3 | 1.15 | 0.036 |
| CMPK2 | 0.72 | 0.037 |
| FAM50A | 0.66 | 0.038 |
| ABCC1 | 1.52 | 0.039 |
| SDSL | 0.57 | 0.040 |
| HLA-B | 0.70 | 0.045 |
| TRIM22 | 0.98 | 0.046 |
| PHLDB2 | 0.89 | 0.047 |
| RRBP1 | 0.52 | 0.048 |
| PSMB8 | 1.36 | 0.049 |
| ANKLE2 | 1.28 | 0.050 |
