# Supplementary material for: MiR-93 is related to poor prognosis in pancreatic cancer and promotes tumor progression by targeting microtubule dynamics
Source: Oncogenesis. 2020 May 4;9(5):43. doi: 10.1038/s41389-020-0227-y (PMC7198506; doi:10.1038/s41389-020-0227-y)
Supplement: Supplementary file 14 — Supplementary table 6 [file 41389_2020_227_MOESM14_ESM.docx]

**Supplementary table 6:** Down-regulated proteins in HPDE-miR-93 cells from the proteomic analysis. Only those proteins detected in 2 or 3 replicas from control cells and undetected in HPDE-miR-93 cells are listed (n=3).

|  | **Count** | |  | | **Count** | |  | | **Count** | |
| --- | --- | --- | --- | --- | --- | --- | --- | --- | --- | --- |
| **Gene name** | **Control** | **+miR-93** |  | **Gene name** | **Control** | **+miR-93** |  | **Gene name** | **Control** | **+miR-93** |
| SMCO2 | 2 | 0 |  | MAGT1 | 2 | 0 |  | FILIP1L | 2 | 0 |
| MPHOSPH10 | 2 | 0 |  | SEPSECS | 2 | 0 |  | ZNF326 | 2 | 0 |
| DEGS1 | 2 | 0 |  | TREX1 | 2 | 0 |  | LRSAM1 | 2 | 0 |
| STX10 | 2 | 0 |  | NECAP2 | 2 | 0 |  | C15orf52 | 2 | 0 |
| DIAPH2 | 2 | 0 |  | DONSON | 2 | 0 |  | PHACTR4 | 2 | 0 |
| CLASP2 | 2 | 0 |  | STARD9 | 2 | 0 |  | GOLGA1 | 2 | 0 |
| IL1A | 2 | 0 |  | DBR1 | 2 | 0 |  | RAB11FIP5 | 2 | 0 |
| AFP | 2 | 0 |  | TRAPPC2L | 2 | 0 |  | DDX19A | 2 | 0 |
| MYZAP | 2 | 0 |  | MYO5B | 2 | 0 |  | STK10 | 2 | 0 |
| TMEM11 | 2 | 0 |  | SLC12A7 | 2 | 0 |  | NNMT | 2 | 0 |
| S1PR1 | 2 | 0 |  | LTN1 | 2 | 0 |  | ARMC6 | 2 | 0 |
| FLT3 | 2 | 0 |  | YIF1A | 2 | 0 |  | MCAT | 2 | 0 |
| CAMLG | 2 | 0 |  | LDHC | 2 | 0 |  | NT5E | 2 | 0 |
| ATXN3 | 2 | 0 |  | USP8 | 2 | 0 |  | GRIPAP1 | 2 | 0 |
| TXNL4A | 2 | 0 |  | ECE1 | 2 | 0 |  | AIDA | 2 | 0 |
| ZFP36L1 | 2 | 0 |  | CSNK1E | 2 | 0 |  | FAT2 | 2 | 0 |
| HMGXB3 | 2 | 0 |  | STXBP1 | 2 | 0 |  | BIN1 | 3 | 0 |
| ADAM15 | 2 | 0 |  | RBPJ | 2 | 0 |  | HLA-E | 3 | 0 |
| CDK13 | 2 | 0 |  | PPIG | 2 | 0 |  | MGAT2 | 3 | 0 |
| LPIN1 | 2 | 0 |  | RIPK1 | 2 | 0 |  | MYSM1 | 3 | 0 |
| NAA30 | 2 | 0 |  | BOP1 | 2 | 0 |  | TBC1D9B | 3 | 0 |
| CUL7 | 2 | 0 |  | DPYSL3 | 2 | 0 |  | SLC25A25 | 3 | 0 |
| TNIP1 | 2 | 0 |  | MRPL23 | 2 | 0 |  | C1orf174 | 3 | 0 |
| ECM1 | 2 | 0 |  | LDLRAP1 | 2 | 0 |  | MCRS1 | 3 | 0 |
| ZBED5 | 2 | 0 |  | THOC7 | 2 | 0 |  | ISCU | 3 | 0 |
| ARHGAP29 | 2 | 0 |  | TDRD7 | 2 | 0 |  | NOP10 | 3 | 0 |
| TSSC1 | 2 | 0 |  | COG1 | 2 | 0 |  | LIMD1 | 3 | 0 |
| GLYATL3 | 2 | 0 |  | ATP6V0A1 | 2 | 0 |  | NCOR2 | 3 | 0 |
| MCTP1 | 2 | 0 |  | ISOC2 | 2 | 0 |  | TRIM21 | 3 | 0 |
| FAT4 | 2 | 0 |  | STARD4 | 2 | 0 |  | MRPS11 | 3 | 0 |
| CERS6 | 2 | 0 |  | WDR92 | 2 | 0 |  | STARD5 | 3 | 0 |
| CCDC141 | 2 | 0 |  | GBP4 | 2 | 0 |  | LOXL2 | 3 | 0 |
| GPR180 | 2 | 0 |  | RBCK1 | 2 | 0 |  | FHAD1 | 3 | 0 |
| FAM129C | 2 | 0 |  | UCK2 | 2 | 0 |  | EPB41L2 | 3 | 0 |
| FRAS1 | 2 | 0 |  | PARP12 | 2 | 0 |  | STAT5B | 3 | 0 |
| VRK2 | 2 | 0 |  | OSBPL2 | 2 | 0 |  | JMJD6 | 3 | 0 |
| ZDHHC23 | 2 | 0 |  | VPS11 | 2 | 0 |  | DNAH17 | 3 | 0 |
| LINC01006 | 2 | 0 |  | TAOK3 | 2 | 0 |  | VIM | 3 | 0 |
| CAPSL | 2 | 0 |  | KIF15 | 2 | 0 |  | ERAP2 | 3 | 0 |
| TSR2 | 2 | 0 |  | CNOT2 | 2 | 0 |  | ISG20 | 3 | 0 |
| UBE2E3 | 2 | 0 |  | DAPP1 | 2 | 0 |  | KIF2C | 3 | 0 |
| SMIM12 | 2 | 0 |  | PAM16 | 2 | 0 |  | MAD1L1 | 3 | 0 |
| THOC1 | 2 | 0 |  | RBM19 | 2 | 0 |  | SRA1 | 3 | 0 |
| SNF8 | 2 | 0 |  | CEP170B | 2 | 0 |  | |  |  |
| TMEM41A | 2 | 0 |  | MAP4K5 | 2 | 0 |  |  |  |  |
| ZNF462 | 2 | 0 |  | NUB1 | 2 | 0 |  |  |  |  |
| ZFP91 | 2 | 0 |  | SH3PXD2B | 2 | 0 |  |  |  |  |
| DDX50 | 2 | 0 |  | INPP4B | 2 | 0 |  |  |  |  |
| SH2D3A | 2 | 0 |  | DAPK3 | 2 | 0 |  |  |  |  |
| MTA3 | 2 | 0 |  | CD55 | 2 | 0 |  |  |  |  |
| MED18 | 2 | 0 |  | STX17 | 2 | 0 |  |  |  |  |
| TBC1D10A | 2 | 0 |  | BPTF | 2 | 0 |  |  |  |  |
| CEP295 | 2 | 0 |  | STX5 | 2 | 0 |  |  |  |  |
| TINAGL1 | 2 | 0 |  | PTPRK | 2 | 0 |  |  |  |  |
