# Supplementary material for: MiR-93 is related to poor prognosis in pancreatic cancer and promotes tumor progression by targeting microtubule dynamics
Source: Oncogenesis. 2020 May 4;9(5):43. doi: 10.1038/s41389-020-0227-y (PMC7198506; doi:10.1038/s41389-020-0227-y)
Supplement: Supplementary file 15 — Supplementary table 7 [file 41389_2020_227_MOESM15_ESM.docx]

**Supplementary table 7.** Top diseases and top 3 networks revealed by IPA analysis according to the proteomic analysis comparing control versus overexpressing miR-93 HPDE cells.

| Top Networks | Score | Focus molecules |
| --- | --- | --- |
| 1. Antigen presentation, protein synthesis, inflammatory response | 46 | 28 |
| 1. Cellular movement, connective tissue disorders, dermatological diseases and conditions | 41 | 26 |
| 1. Cellular assembly and organization, cellular function and maintenance, cell morphology | 20 | 16 |

| Top diseases and disorders | p-value range | No. of proteins |
| --- | --- | --- |
| Cancer | 3,60E-02 – 4,92E-05 | 213 |
| Gastrointestinal disease | 3,60E-02 – 4,92E-05 | 201 |
| Organismal injury and abnormalities | 3,91E-02 – 4,92E-05 | 216 |
